# Thermal Phase Behavior, Structure, Acidity, and Quantum Chemical Insights into Novel Hydroxylamine Hydrochloride (HACl)-Methanesulfonic Acid (MSA) Deep Eutectic Solvents

**DOI:** 10.1021/acsomega.6c06790

**Published:** 2026-10-22

**Authors:** Hosein Ghaedi, Payam Kalhor, Cristian Serrano Araya, Richard Telford, Yousef Ghorbani

**Affiliations:** † College of Health and Science, School of Natural Sciences, Department of Chemistry, Joseph Banks Laboratories, 4547 University of Lincoln, Green Lane, Lincoln, Lincolnshire LN6 7DL, U.K.; ‡ School of Chemistry, 7423 University of Southampton, Southampton SO17 1BJ, U.K.; § School of Chemistry and Biosciences, Faculty of Health and Social Care, 1905 University of Bradford, Richmond Road, Bradford BD7 1DP, U.K.

## Abstract

The present study undertakes the first characterization of hydroxylamine hydrochloride (HACl)-methanesulfonic acid (MSA) deep eutectic solvents (DESs), a new generation of sustainable, dual-functional solvents with exceptionally strong acidic and inherently reduced properties. Employing FTIR, Raman, and NMR spectroscopy, the nature of HACl-MSA DESs’ molecular interactions was probed with varying molar ratios between 1:4 and 1:8. Differential scanning calorimetry (DSC) revealed composition-dependent phase behavior, transitioning from an amorphous glass-forming liquid at 1:4 (*T*_g_ = −89 °C, peak at 85.87 °C) to congruent melting at 1:5 (*T*_m_ = +5.43 °C). For 1:6 to 1:8 formulations, invariant eutectic behavior was observed with a shared solidus transition at about −53.80 °C and a clear melting range ending between −45.91 and −39.21 °C. Spectroscopic analyses confirmed the formation of a homogeneous, single-phase protic DES characterized by a robust, shielded hydroxylammonium core ([NH_3_OH]^+^) at 9.87–9.88 ppm and a dynamic acid proton pool that shifts downfield from 13.19 to 13.63 ppm. This shifting resonance directly reflects a tunable macroscopic acidity driven by the expansion of the MSA-MSA hydrogen-bonded network. Quantitative Hammett measurements (*H*_0_), determined via UV–vis spectroscopy, demonstrated significant acidity, reaching −6.116 at the 1:8 ratio. Density functional theory (DFT) analysis also confirmed the results, revealing a greater internal thermodynamic stability for the 1:5 ratio with a |Δ*E*_int_| = 4.280 eV. However, conceptual DFT descriptors, including a smaller energy gap (7.324 eV), a larger global softness value of 0.137 eV^–1^, and a more negative chemical potential (−4.506 eV), highlight the 1:4 ratio as a promising theoretical candidate for further evaluation in metal coordination processes. While these electronic metrics suggest a highly polarizable microenvironment, direct extraction yields, leaching kinetics, viscosity, and selectivity require future experimental validation. Through correlations established between molecular structure, frontier orbital analysis, and macroscopic Brønsted acidity trends, HACl–MSA DESs are highlighted as promising candidate platforms for potential application in hydrometallurgical leaching processes. However, definitive claims regarding selective metal extraction, long-term stability, recyclability, and environmental sustainability require comprehensive future experimental evaluation.

## Graphical abstract

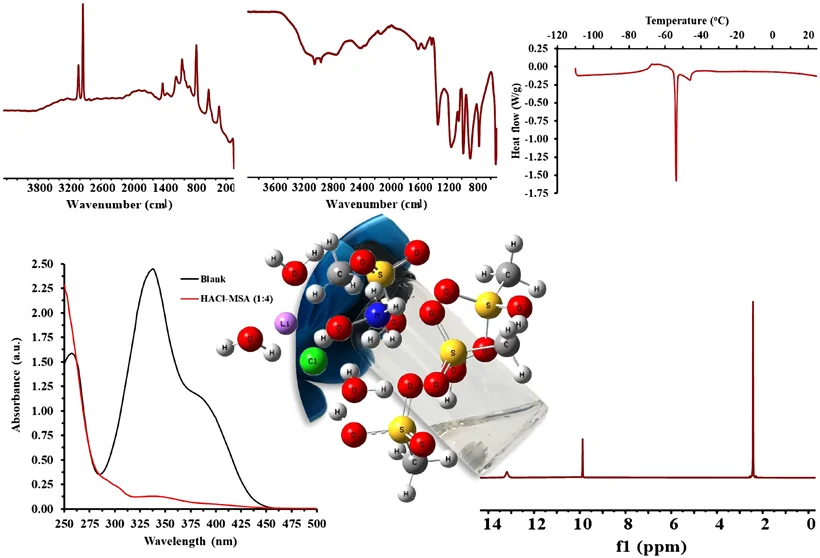

## 1 Introduction

The global demand for metals, particularly critical and transition elements, necessitates the development of sustainable and efficient extraction methodologies. The secure supply of these materials, which include elements like lithium, cobalt, nickel, and various rare earth elements, is a geo-strategic priority for economic stability and the green energy transition, particularly across the United Kingdom and Europe. These elements are indispensable for key technologies such as electric vehicles, wind turbines, and advanced electronics, underpinning the continent’s commitment to decarbonization and technological sovereignty.

Traditional hydrometallurgical and pyrometallurgical processes are often associated with high energy consumption, substantial waste generation, and the use of volatile, toxic organic solvents or corrosive acids. Pyrometallurgy, which relies on high-temperature smelting, is effective for high-concentration ores but is energy-intensive and produces significant gaseous emissions (e.g., SO_2_) and solid slag waste.^1–4^ Hydrometallurgy, while operating at lower temperatures, often utilizes strong, environmentally harsh reagents like cyanide or concentrated sulfuric acid, leading to large volumes of wastewater that require complex treatment.^5–7^ This reliance on conventional, environmentally burdensome methods presents a major hurdle to achieving a circular economy and has fueled the search for sustainable alternatives, giving rise to the field of advanced metal processing, which utilizes novel, often nonaqueous solvents for metal separation and recovery.^8,9^ Among these emerging solvents, deep eutectic solvents (DESs) have garnered significant attention due to their unique properties, including low volatility, nonflammability, low toxicity, and highly tunable physicochemical characteristics.^10–16^ The DESs are generally regarded as safer and more cost-effective alternatives to conventional ionic liquids, offering comparable solvation power while minimizing environmental and safety hazards associated with volatile organic compounds.^9,17^

DESs are typically formed by mixing a hydrogen bond donor (HBD) with a hydrogen bond acceptor (HBA), resulting in a eutectic mixture with a melting point significantly lower than its individual components. The strong hydrogen-bonding interactions between the components are responsible for disrupting the crystal lattice energy of the starting materials, thereby stabilizing the liquid phase at or near ambient temperatures. Protic DESs, formed by combining a Brønsted acid and a base, are particularly valuable in metallurgical applications as they offer inherent acidity and high dissolving power crucial for leaching metal oxides and sulfides. The hydroxylamine hydrochloride (HACl) and methanesulfonic acid (MSA) system represents a highly promising class of protic DES. HACl is a readily available, low-cost hydrogen bond acceptor, and importantly, it functions as a potent, nonmetal-contaminating reducing agent due to the hydroxylamine moiety (NH_2_OH). MSA is selected as the HBD because it is a strong, nonoxidizing Brønsted acid known for its low corrosivity, high thermal stability, and excellent biodegradability.^18–23^ The combination of these two components creates a strongly Brønsted-acidic, manageable solvent platform designed for leaching, dissolution, and reductive metal recovery processes where the simultaneous application of acidity and a reducing environment is required. However, specific performance attributes regarding overall process sustainability, solvent stability, and operational efficiency remain to be systematically validated.

This work is the first study to systematically investigate the HACl-MSA system. A precise, molecular-level understanding of how component stoichiometry controls the solvent’s structure, the degree of proton transfer, the hydrogen-bonding network, and ultimately its bulk acidity remains a critical knowledge gap. The nature of the anion and cation interactions fundamentally dictates the DES’s ability to dissolve specific metal species. For potential metal processing applications, the ability to fine-tune the solvent’s proton environment is critical for influencing the dissolution behavior and speciation of metal ions. While this tuneability suggests promising avenues for customized leaching, specific claims regarding metal extraction selectivity require dedicated future experimental testing. This study aims to bridge this critical gap by synthesizing and systematically characterizing HACl-MSA DESs across an expansive molar range (1:4 to 1:8, HACl/MSA). This broad range allows for the investigation of the full transition from a hydrogen-bond-stabilized eutectic melt to an MSA-dominated highly acidic system.

A comprehensive analytical approach utilizing differential scanning calorimetry (DSC), Fourier-transform infrared (FTIR) spectroscopy, Raman spectroscopy, and nuclear magnetic resonance (^1^H NMR) spectroscopy is employed to elucidate the thermal phase behavior and correlate changes in molecular structure and dynamic proton networks across varying HACl/MSA molar ratios. Furthermore, the true acidity of the DESs is quantitatively assessed using the Hammett acidity function (*H*_0_) by employing ultraviolet–visible (UV–vis) spectroscopy. The present experimental studies are combined with density functional theory (DFT) calculations to establish a basic quantum chemical framework of the observed behaviors. The electronic driving forces responsible for the reactivity of the system are elucidated through the evaluation of global reactivity descriptors such as chemical potential, global softness, and electrophilicity alongside frontier molecular orbital (HOMO–LUMO) distributions. Ultimately, this study sets up a strong relationship between structure and property, linking spectroscopic fingerprints and computational electronic profiles to bulk acidity. This provides a rational foundation for evaluating HACl–MSA DESs as candidate solvents for future hydrometallurgical applications. However, claims regarding operational extraction yields, leaching selectivity, recyclability, solvent stability, and life-cycle sustainability represent potential application directions that require thorough future experimental validation.

## 2 Experimental Section

### 2.1 Chemicals

The MSA (≥99%), 4-nitroaniline (≥99%), and 2,4-dinitroaniline (98%) were received from Sigma-Aldrich, sourced from Germany, Spain, and China, respectively. The HACl (99%) and dimethyl sulfoxide (DMSO)-*d*_6_ (≥99.85%) were obtained from Thermo Scientific, sourced from China and Switzerland, respectively. Absolute ethanol (≥99.8) was supplied by Fisher Chemical (UK). The NMR tubes (500 MHz, OD: 7 mm, length: 7 in., thin wall: 0.43 mm) were purchased from Wilmad-Labglass (Vineland). Quartz cuvettes (wavelength range: 190∼2500 nm, path length: 10 mm, volume: 3.5 mL) were received from Best Quality (India).

### 2.2 Preparation of DESs

The DES used in this study is HACl-MSA in various molar ratios of 1:4 to 1:8. The required amount of HACl and MSA was mixed and heated on a hot plate at a temperature of 60 to 80 °C (depending on the ratio) until a uniform liquid was obtained. The formed DESs were kept in tight bottles to prevent any contamination from the outside atmosphere. The DESs were used without any further purification. Table 1 presents the HBA/HBD molar ratios, mole fractions, mass fractions, and average molar masses. Figure S1 shows photos of formulated DESs from molar ratios of 1:1 to 1:8.

**Table 1:** Stoichiometric and Physical Properties of the HACl–MSA DES Series

DES abbreviation (molar ratio)	mole fraction HACl (*x*_HACl_)	mole fraction MSA (*x*_MSA_)	mass fraction HACl	mass fraction MSA	molar mass of DES(g/mol)^a^
HACl-MSA (1:4)	0.200	0.800	0.153	0.847	90.79
HACl-MSA (1:5)	0.167	0.833	0.126	0.874	91.67
HACl-MSA (1:6)	0.143	0.857	0.108	0.892	92.31
HACl-MSA (1:7)	0.125	0.875	0.094	0.906	92.78
HACl-MSA (1:8)	0.111	0.889	0.083	0.917	93.15

a The molar mass of DES (g/mol) was calculated according to Ghaedi et al.^24^

### 2.3 Differential Scanning Calorimetry (DSC)

Thermal transitions were evaluated using a TA Instruments DSC. Liquid DES samples (1.5–3.0 mg) were hermetically sealed in aluminum pans under a nitrogen purge. Samples were heated to 50 °C at 5 °C/min and held for 10 min to erase thermal history, cooled to −110 °C at 1 °C/min and held for 30 min, and then reheated to 25 °C at 2 °C/min. Transition temperatures (glass transition temperature (*T*_g_), crystallization temperature (*T*_c_), and melting point (*T*_m_)) were determined from the reheating thermograms.

### 2.4 Determination of Hammett Acidity Function (*H*_0_)

For measuring the Brønsted acidity of pure DESs and their aqueous solutions, the Hammett acidity function (*H*_0_) was applied. The *H*_0_ values were determined following the unified Hammett equation:^22,25,26^

1 $$H_{0}={pK}_{a}+\log\left(\frac{\left[I\right]}{\left[{\mathrm{IH}}^{+}\right]}\right)$$

where [*I*] and [*IH* ^+^] represent unprotonated and protonated indicator concentrations (or percent), respectively, and the ratio [*I*]/[*IH* ^+^] = (*A*/(*A*_max_ – *A*)) was obtained from absorbance measurements. *A*_max_ is the maximum absorbance of the completely unprotonated indicator in reference media, and *A* is the measured absorbance of the indicator in the sample at λ_max_. This formulation assumes zero absorbance contribution from the protonated form (*IH* ^+^) at the analytical wavelength. The method accounts for contributions from both unprotonated and protonated species and thus is applicable over a range of acidity in aqueous and pure DES systems.

For aqueous DES solutions, the indicator selected was 4-nitroaniline with a p*K*_a_ value of 0.99, and the absorbance was measured at 380 nm. Aqueous DES solutions were prepared at a concentration of 40 mmol/L. A stock solution of 0.6 mmol/L 4-nitroaniline was prepared by dissolving 0.0083 g of 4-nitroaniline in 100 mL of deionized water using a 100 mL volumetric flask. The mixture was heated gradually to 50 °C under stirring to fully dissolve, and then cooled to room temperature and stored in an amber bottle and kept in order to prevent photodegradation. A 0.1 mmol/L solution of 4-nitroaniline was prepared by diluting 0.833 mL of stock 0.6 mmol/L to 5 mL with deionized water in a 5 mL volumetric flask to a concentration of 0.1 mmol/L (13.812 mg/L). The 0.1 mmol/L solution was used as the blank for *A*_max_ determination and for preparing the DES sample solutions. The blank solution, 3 mL of 0.1 mmol/L 4-nitroaniline in deionized water, was measured to determine *A*_max_. The spectrophotometer was baselined with 3 mL of deionized water, and the blank was scanned from 250 to 500 nm, which gave *A*_max_ at 380 nm. For each DES sample, a 40 mmol/L DES and 0.1 mmol/L 4-nitroaniline solution was prepared (2.5 mL of 40 mmol/L stock DES + 0.5 mL of 0.6 mmol/L stock 4-nitroaniline, total 3 mL). The baseline of the DES sample was set with 3 mL of 40 mmol/L DES solution (without 4-nitroaniline) to compensate for DES absorbance. The sample was then read at 380 nm for the absorbance (*A*). In addition, the *H*_0_ was determined for aqueous solutions of HACl-MSA (1:8) at varying concentrations: 100, 500, 1000, and 1500 mmol/L to investigate the concentration-dependent effects on acidity.

For pure DESs, 2,4-dinitroaniline was employed as the indicator (p*K*_a_ value of −4.53), with absorbance measured at approximately 338–342 nm depending on the DES composition. A stock solution of 2,4-dinitroaniline was prepared by dissolving 25 mg (0.025 g) of 2,4-dinitroaniline in 5 mL of ethanol in a 5 mL vial. The mixture was slowly warmed to 40–50 °C for 2 min under stirring in order to completely dissolve, then cooled at room temperature, and stored in an amber bottle in a refrigerator in order to prevent photodegradation. Individual measurement of *A*_max_, the absorbance of the fully unprotonated indicator, was performed by preparing a 3.5 mL working solution. This was achieved by transferring 0.022 mL (22 μL) of the prepared stock solution into a new 5 mL vial and adding 3.478 mL of ethanol, thus attaining a concentration of approximately 1.7 × 10^–1^ mmol/L. Subsequently, 3 mL was transferred from this into a new 3 mL quartz cuvette, and absorbance was measured at 338 nm. The baseline was adjusted using 3 mL of ethanol (no indicator) to account for the inherent absorbance. The sample absorbance (*A*) was then found by preparing a 3.5 mL working solution by combining 0.022 mL (22 μL) of the prepared stock solution with 3.478 mL of the pure DESs in various ratios to provide a concentration of approximately 1.7 × 10^–1^ mmol/L. This mixture was then heated and stirred at 40–50 °C for 2 min to enhance dissolution, particularly of the viscous DESs. The spectrophotometer’s baseline was established with 3 mL of the pure DES (no indicator) in a 3 mL quartz cuvette to account for any natural absorbance. The working solution (3 mL transferred from the 3.5 mL vial into a new 3 mL quartz cuvette) was scanned, and the absorbance (*A*) was read at the wavelength of maximum absorption. All measurements were carried out at room temperature, and quartz cuvettes were rinsed with deionized water and acetone between runs to prevent contamination.

The absorbance measurement uncertainty *u*(*A*) was evaluated directly as the root mean square error (RMSE) across *n* = 3 independent spectrophotometric replicates over the 250–500 nm spectral window to capture instrument noise, background scatter, and baseline fluctuations. Standard uncertainties *u*(*H*_0_) were calculated using nonlinear error propagation via eq 2. Furthermore, because measurements in pure DES media rely on literature indicator p*K*_a_ values and are subject to matrix effects (variations in molar extinction coefficients, local dielectric constant, and solvatochromism), the reported *H*_0_ values represent operational, indicator-dependent acidity metrics.

The *u*(*H*_0_) can be calculated as follows

2 $${u(H}_{0})=\sqrt{\left[u^{2}\left({pK}_{a}\right)+{\left(S\times u\left(A\right)\right)}^{2}\right]}$$

where *u*(*A*) is the absorbance measurement uncertainty, and *S* is the sensitivity factor derived from the Hammett equation

3 $$S=\frac{1}{\ln\left(10\right)}\left|\frac{1}{A}+\frac{1}{A_{\max}-A}\right|$$

This uncertainty propagation model was applied across all compositions to determine the final combined standard uncertainty associated with each calculated *H*_0_ value.

### 2.5 Characterizations

The DSC measurements were performed using a Discovery 2500 DSC equipped with a refrigerated cooling system (RCS) 120 (Waters Materials Sciences, a division of Waters Corporation). Tzero hermetic aluminum pans were used for the measurements and sealed with the corresponding pin-holed lids. The FTIR spectrometer (Bruker α spectrometer with Platinum-ATR module, Germany) was used to identify the functional groups of DES and hydrogen-bond interactions in the wavelength range from 400 to 4000 cm^–1^. Raman tests were performed using a Bruker SENTERRA II Raman Microscope at room temperature, and the spectra were recorded with an excitation laser wavelength of 532 nm in a range from 100 to 4000 cm^–1^. The laser power, integration time, coadditions, and objective were fixed at 20 mW, 1000 ms, 30 scans, and 4×, respectively. An aluminum metal substrate was employed for sample deposition and measurement to avoid background interference and to provide sample presentation uniformity. Data collection was performed with OPUS software, while analysis and processing were performed with MestReNova software. NMR (^1^H) spectra were obtained on a Bruker AM 400 MHz NMR instrument equipped with a 5 mm PABBO BB/19F-1H/D Z-GRD probe (Z119470/0185). The experiment was carried out at 298 K employing DMSO-*d*_6_. Samples were prepared by dissolving 0.23 mL of DES or pure MSA in 0.47 mL of DMSO-*d*_6_ and 40 mg of pure HACl in 0.7 mL of DMSO-*d*_6_, using an analytical balance and a micropipette for the measurements. The spectral width, acquisition time, and relaxation delay were adjusted to 10 kHz, 3.28 s, and 1.0 s, respectively. All NMR spectra were acquired, processed, and analyzed using TopSpin 4.4.1 software (Bruker Biospin) to ensure accurate spectral acquisition and interpretation over the examined molar ratios. The Brønsted acidity of the pure DESs and their aqueous solutions in terms of *H*_0_ was measured through UV–vis spectroscopy (SPECORD 210 PLUS, Analytik Jena, Germany) using 4-nitroaniline and 2,4-dinitroaniline as indicators. The spectra were scanned between 250 and 500 nm at room temperature.

### 2.6 Density Functional Theory (DFT)

Density functional theory (DFT) is employed to elucidate the molecular interactions and physicochemical properties of HACl-MSA DESs, specifically focusing on hydrogen-bonding networks and potential protonation effects observed in experimental vibrational spectroscopy. DFT simulations provide critical insights into structure–property relationships that govern DES formulation and stability. All calculations were performed using the GAUSSIAN 16 software package. Molecular geometries of HACl, MSA, and their DES complexes were optimized using Becke’s three-parameter exchange functional combined with the Lee–Yang–Parr gradient-corrected functional (B3LYP), coupled with the 6–311++G­(d,p) basis set and Grimme’s D3 dispersion correction with Becke-Johnson damping (empiricaldispersion = gd3bj) to accurately model hydrogen-bonding and van der Waals interactions essential for the HACl-MSA DES. The 6–311++G­(d,p) basis set includes diffuse and polarization functions to describe the electronic structure of hydrogen-bonded and ionic systems effectively. Initially, the geometries of individual HACl and MSA molecules were optimized. Subsequently, multiple configurations of the HACl-MSA DES at various molar ratios (e.g., 1:4 and 1:5, HACl/MSA) were optimized, guided by electrostatic potential (ESP) maps to identify key interaction sites. Interaction energies were corrected using the counterpoise method to mitigate basis set superposition errors (BSSE).^15^ The absolute interaction energy (|Δ*E*_int_|) for DES formation was calculated as follows

4 $$\left|\Delta E_{\mathrm{int}}\right|{=E}_{\mathrm{formation}}-\sum_{i=1}^{n}E_{\mathrm{fragment}\left(i\right)}$$

where *E*_formation_ represents the (counterpoise-corrected) energies of DES structures. *E*_fragment (*i*)_ is the energy of individual fragment *i*, calculated within the same formed DES, and *n* is the number of fragments in the formed DES.

The highest occupied molecular orbital (HOMO) and lowest unoccupied molecular orbital (LUMO) are collectively referred to as the frontier molecular orbitals (FMOs). These orbitals play a critical role in determining the chemical reactivity and stability of molecules. The HOMO represents the ability of a molecule to donate electrons (nucleophilicity), while the LUMO reflects its ability to accept electrons (electrophilicity). The energy difference between these orbitals, known as the HOMO–LUMO gap, is often used as an indicator of molecular stability: smaller gaps generally imply higher chemical reactivity and lower kinetic stability. Based on the FMO energies, a set of global reactivity descriptors was calculated to further characterize the electronic structure and reactivity profile of each system. These include ionization potential (IP), electron affinity (EA), chemical potential (μ), hardness (η), softness (*S*), electrophilicity index (ω), and electronegativity (χ). The calculations were performed using Koopmans’ theorem as a working approximation, wherein the orbital energies are treated as estimates of ionization potential and electron affinity. The descriptors were computed using the following equations

5 $$IP=-E_{\mathrm{HOMO}}$$

6 $$EA=-E_{\mathrm{LUMO}}$$

7 $$\mu \approx -\left(\frac{IP+EA}{2}\right)\approx \frac{E_{\mathrm{LUMO}}+E_{\mathrm{HOMO}}}{2}$$

8 $$\eta \approx \frac{IP-EA}{2}\approx \frac{E_{\mathrm{LUMO}}-E_{\mathrm{HOMO}}}{2}$$

9 $$S\approx \frac{1}{IP-EA}\approx \frac{1}{E_{\mathrm{LUMO}}-E_{\mathrm{HOMO}}}$$

10 $$\omega =\mu^{2}/2\eta$$

11 χ=−μ

These values provide a comprehensive framework for interpreting the stability and reactivity trends across HACl–MSA DESs.

## 3 Results and Discussion

### 3.1 Thermal Behavior and Phase Transitions of DESs

The thermal behavior of the HACl–MSA series demonstrates a strong dependence on chemical stoichiometry, evolving from fully vitrifying system dynamics to congruent crystallization and multistage eutectic-like phase transitions. Figure 1 illustrates the DSC thermograms recorded across the investigated composition range (1:4 to 1:8 molar ratios).

**Figure 1:**
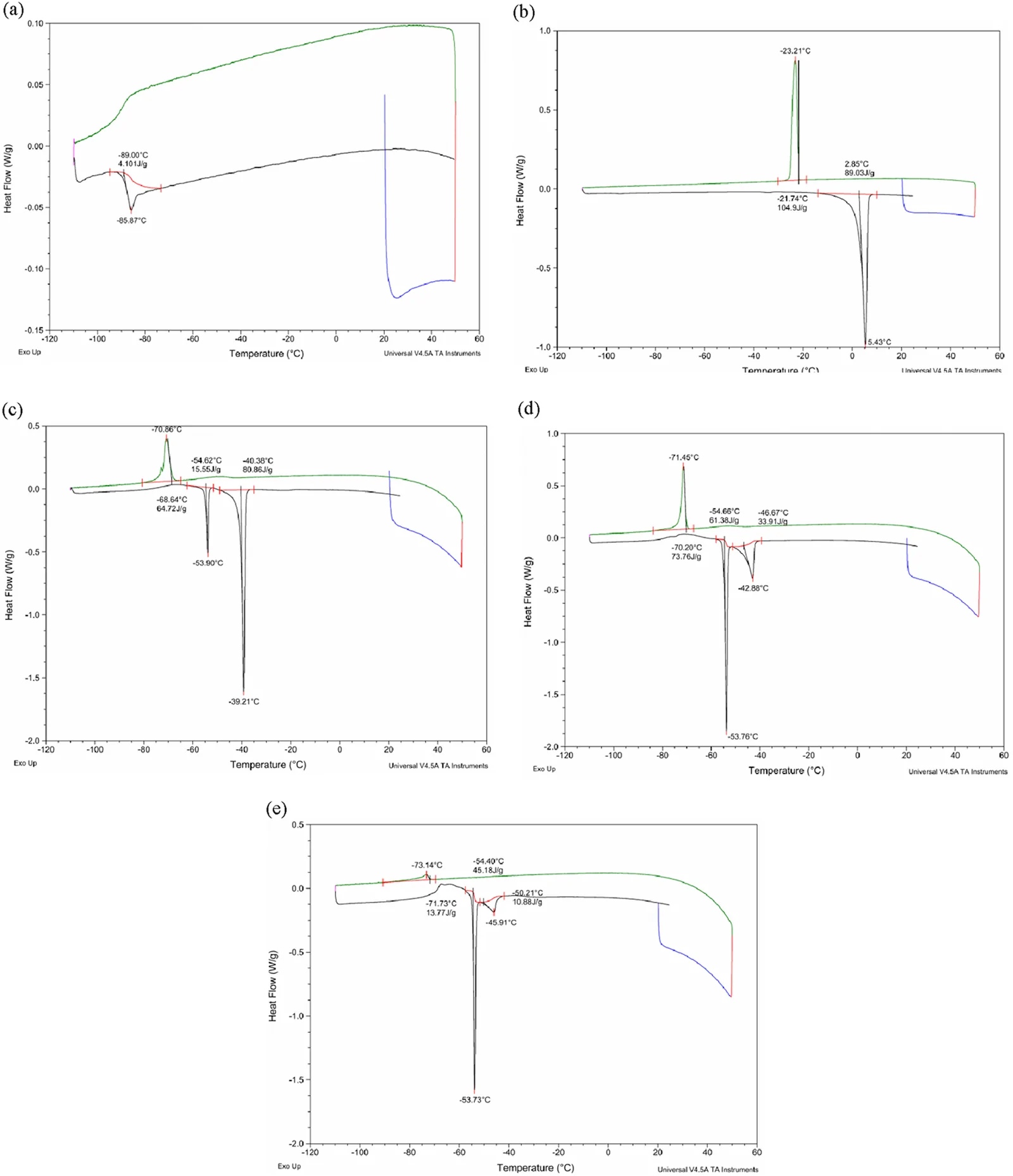
DSC curves of (a) HACl-MSA (1:4), (b) HACl-MSA (1:5), (c) HACl-MSA (1:6), (d) HACl-MSA (1:7), and (e) HACl-MSA (1:8).

The HACl–MSA (1:4) formulation exhibits classic glass-forming behavior. Upon cooling down to −110 °C, crystallization is completely bypassed, and reheating reveals a distinct glass transition step at *T*_g_ = −89 °C accompanied by a minor endothermic enthalpy relaxation peak at −85.87 °C (Δ*H* = 4.101 J/g). The absence of crystallization exotherms or melting endotherms indicates that the 1:4 ratio forms an amorphous, highly interconnected supramolecular network.

In contrast, increasing the acid content to 1:5 (HACl–MSA 1:5) yields a congruently melting phase profile. During cooling, a sharp crystallization exotherm manifests with an onset at −21.74 °C and a peak at −23.21 °C (Δ*H*_c_ = 89.03 J/g). Upon reheating, a well-defined melting endotherm is observed starting at +2.85 °C and peaking at *T*_m_ = +5.43 °C (Δ*H*_m_ = 104.90 J/g). For HBD-rich formulations from 1:6 to 1:8, the thermal profile shifts toward significantly lower transition temperatures. These compositions exhibit pronounced supercooling during cooling, with crystallization exotherms manifesting between −70.86 and −73.14 °C. Upon reheating, all three compositions display a dual endothermic melting envelope rather than a single transition. The first endothermic peak occurs at an invariant temperature of −53.73 to −53.90 °C across the series, designating a shared solidus transition. The second endothermic peak (*T*_m,2_) corresponds to the liquidus boundary and shifts systematically to lower temperatures from −39.21 °C (1:6) to −42.88 °C (1:7) and −45.91 °C (1:8), reflecting the progressive dissolution of excess MSA into the liquid matrix. While the observed depression in transition temperatures (Δ*T* > 145 C relative to pure HACl) indicates strong intermolecular interactions and confirms that all formulations remain fluid at room temperature, a formal thermodynamic analysis of the ideal liquidus (using the Schröder–van Laar equation and activity coefficients) would be required to quantitatively assess the extent of negative deviation from ideal-liquid behavior.

In summary, the thermal characterization of the HACl–MSA DES platform establishes a clear, stoichiometry-driven phase evolution: transitioning from an amorphous glass-forming liquid at 1:4, to a congruently melting system at 1:5, and finally to a eutectic-like regime across 1:6–1:8 compositions defined by an invariant solidus and systematic liquidus depression. This compositional flexibility affords broad subzero liquid stability, preventing premature phase separation and maintaining a fluid processing window for low-temperature applications.

### 3.2 FTIR Spectroscopy Analysis

FTIR spectra of HACl-MSA DESs across molar ratios of 1:4 to 1:8 show how interactions between molecules change, as indicated by the major peaks in the vibrational spectra. The FTIR spectra of the DESs and their individual components are presented as stacked and individually separated spectra in Figures 2 and S2, respectively, with the corresponding peak positions summarized in Table S1.

**Figure 2:**
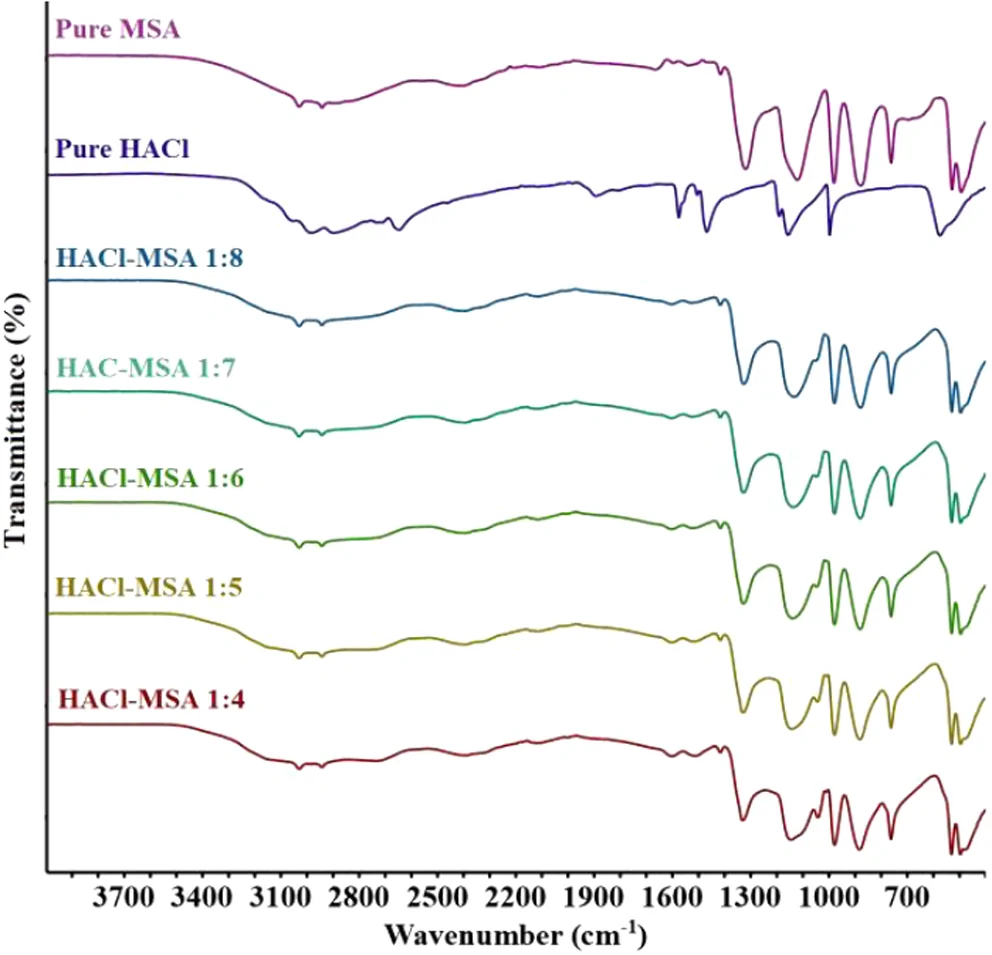
FTIR spectra of pure HACl, pure MSA, and HACl-MSA DES in various molar ratios over the full wavenumber range (4000–400 cm^–1^).

Comparison of the FTIR spectra of the DESs to the FTIR spectra of pure MSA (3030, 2942, 2408, 1665.5, 1598, 1539.5, 1415, 1319, 1121.5, 980.8, 876.8, 762.6, 528, 493.5 cm^–1^)^27–29^ and pure HACl (3054.52, 2985.19, 2893.43, 2734.38, 2711.95, 2461.15, 1894.29, 1576.20, 1504.83, 1468.13, 1190.81, 1156.15, 997.10, 575.02 cm^–1^)^30,31^ will indicate the primary molecular changes that occur in the formation of the eutectic mixture.

In the range of 3000–3500 cm^–1^, a peak observed at 3136.08 cm^–1^ (ratio of 1:4) has been assigned to N–H and O–H stretching vibrations involving the hydroxylammonium cation ([NH_3_OH]^+^). It is higher than that for HACl at 3054.52 cm^–1^ due to changes in the hydrogen-bonding environment resulting from interaction between HACl molecules and sulfonic acid groups of MSA. With an increase in the ratio of MSA components from 1:6 to 1:8, this peak is overshadowed by the broad major peak due to MSA vibrational contributions, indicating dilution of HACl signals and a shift toward an MSA-dominated ionic environment.

A peak observed at 3030.05 cm^–1^, found consistently in every ratio, can be attributed to the C–H stretching vibration of the MSA methyl group (CH_3_). Even this peak holds strongly, becoming slightly sharper at the 1:8 ratio. The observed sharpening of the peak indicates better uniformity in the vibrational environment and less steric hindrance due to the presence of excess MSA, which makes the resulting ionic liquid more homogeneous. Similarly, the observed peak at 2942.37 cm^–1^, consistent with pure MSA at 2942 cm^–1^, also persists through every ratio. The greater intensity found in the 1:8 ratio suggests that the MSA component is dominant.

The major diagnostic changes appear in the S=O stretching region. The asymmetric S=O stretching vibration changes slightly from 1331.51 cm^–1^ at the 1:4 molar ratio to 1327.43 cm^–1^ at the 1:8 molar ratio, with a slight increase in intensity. More significant is the displacement of the symmetric S=O stretch, shifting from 1145.95 cm^–1^ (1:4) to 1131.68 cm^–1^ (1:8), a red shift of ∼14 cm^–1^, with an enhanced intensity. The latter reflects a reduction in S=O bond strength due to stronger, more directional hydrogen-bonding interactions with the hydroxylammonium protons, while the enhanced intensity is indicative of increased participation of sulfonate residues in the hydrogen-bonding network. These changes indicate a more saturated and stronger local hydrogen-bonding region in high ratios of MSA and a concomitant transition toward an ionic, MSA-rich environment.

At low wavenumber, the shoulder corresponding to HACl at ∼477 cm^–1^ (C–N–O bending) becomes less distinct beyond the 1:5 ratio, indicating further dilution and integration of HACl with the acidic MSA system.

To gain deeper structural insight into these vibrational dynamics, the FTIR behavior of the HACl–MSA system was directly evaluated alongside the inorganic ammonium sulfate (AS)–MSA highly acidic platform across comparable molar ratios (1:5 to 1:9).^26^ In both systems, the aliphatic backbone of MSA acts as an inert spectator, with the C–H stretching vibrations remaining completely invariant across all compositions. Furthermore, both platforms exhibit a systematic red shift in the asymmetric S=O stretching mode as the MSA content expands, reflecting a shared mechanism where excess MSA polarizes the sulfonate group.

However, the symmetric S=O stretching region reveals key operational differences between the two networks. In AS–MSA, the sulfonate band shifts steadily from 1152 to 1141 cm^–1^, whereas HACl–MSA undergoes a more pronounced red shift down to 1131.68 cm^–1^ (Δ*ν* = −14 cm^–1^). This larger magnitude shift indicates that the localized chloride-mediated hydrogen-bonding manifold (S–OH···Cl^–^) and hydroxylammonium cation in HACl–MSA induce stronger polarization of the S=O bonds than the sulfate/bisulfate network of AS–MSA. Additionally, the two systems display contrasting trends in their sulfate/cation hydrogen-bonding environments. In AS–MSA, the sulfate band exhibits its maximum red shift relative to pure AS (1055.21 cm^–1^) at the 1:5 ratio (1035.84 cm^–1^), before shifting slightly back toward higher wavenumbers (1046.04 cm^–1^ at 1:9) as the excess MSA matrix dilutes individual salt interactions. In contrast, the [NH_3_OH]^+^ core in HACl–MSA is rapidly subsumed into the broad MSA absorption envelope without structural relaxation. This comparative analysis suggests that while both systems achieve high acidity through MSA matrix domination, HACl–MSA maintains tighter, more localized cationic coordination alongside its dynamic acid fluidity.

Taken together, the FTIR results demonstrate that the HACl-MSA DES system transitions from a hydrogen-bonding-dominant to an ionic structure with an increased MSA ratio. The enhancement of the S=O hydrogen-bond interactions and the suppression of the HACl modes with the prominence of the MSA modes indicate the development of a tunable, highly acidic solvent.

### 3.3 Raman Spectroscopy Analysis

Raman spectroscopy provides a complement to FTIR by detailing the evolution of symmetry changes in the MSA framework as well as the transition from a crystalline solid of HACl salt to a liquid state. The combination of the two approaches distinguishes between the tightening of local hydrogen bonds in the FTIR and the general structural transformation in the Raman. Figures 3 and S3 present the Raman spectra of the DESs and their constituent compounds as stacked and individually separated spectra, respectively. Table S2 summarizes the corresponding peak positions.

**Figure 3:**
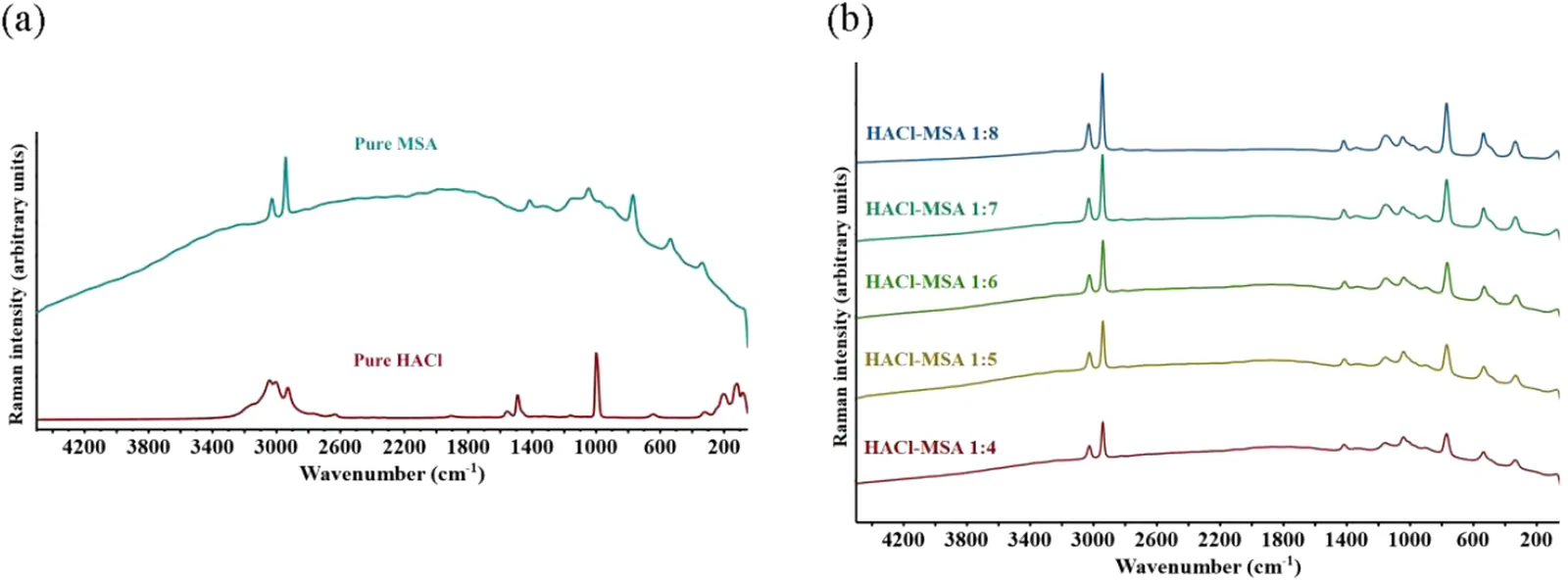
Raman spectra of pure HACl and MSA, and HACl-MSA DESs in various molar ratios (a, b).

Figure 3a shows the Raman spectra of pure HACl and MSA. These data establish a baseline for identifying the molecular changes in the DES. A comparative analysis against the characteristic bands of pure MSA (3357, 3246, 3027, 2943, 1416, 1047, 769.5, 535, 336 cm^–1^)^32,33^ and pure HACl (3006, 2929.5, 2632.5, 1906.5, 1557, 1492.5, 1162.5, 999, 643.5, 321, 199.5, 118.5, 84 cm^–1^) elucidates the fundamental structural modifications and molecular reorganizations triggered by the formation of the eutectic mixture.

The Raman spectra of DESs are illustrated in Figure 3b. In the 3200–3400 cm^–1^ region, broad O–H stretching bands observed at 3343.5 and 3226.5 cm^–1^ (1:4 ratio) correspond to the MSA hydroxyl groups and the hydroxylammonium cation ([NH_3_OH]^+^). For the 1:8 ratio, the bands shift to 3363 and 3244 cm^–1^ with a reduction in intensity. reorganization. The Raman data provide a unique contrast to FTIR findings in the hydroxyl region, where FTIR shows a simple increase in intensity; the Raman signal exhibits marked broadening. This effect underscores a transition toward greater environmental heterogeneity, reflecting a broad distribution of hydrogen-bonding geometries and the establishment of a sophisticated, multicentered proton network at the 1:8 ratio. The C–H stretching corresponding to the methyl group, which occurs between 3027–3031.5 cm^–1^ and 2941.5–2944.5 cm^–1^, retains its correspondence with pure MSA, but increased intensity denotes the greater MSA-based framework at higher molar concentrations.

The sulfonyl environment shows distinct ratio-dependent behavior. The asymmetric S=O stretching vibration at 1335 cm^–1^ for the ratio of 1:4 shifts to a higher wavenumber of about 1339.5 cm^–1^ as the ratio changes to 1:8, and the intensity increases with the concentration of MSA. A significant diagnostic peak for the HBA is the N–O stretching band of the hydroxylammonium cation, observed at 1158 cm^–1^ (1:4). The systematic red shift to 1153.5 cm^–1^ at the 1:8 ratio, despite the lower relative concentration of HACl, indicates that the hydroxylammonium cation undergoes stronger hydrogen-bonded coordination within the HBD-rich acid matrix.

In the 700–900 cm^–1^ region, the C–S stretching at 769.5 cm^–1^ and the S–OH stretching near 904.5 cm^–1^ show marked broadening. This broadening serves as a unique diagnostic signature in Raman that complements the FTIR data by reflecting the diverse coordination states of the sulfonate groups. These states, which range from free to heavily hydrogen-bonded, are less distinguishable in the dipole-driven FTIR spectra. Such spectral behavior indicates the formation of an ″acidic proton pool,″ where the MSA molecules exist in a variety of hydrogen-bonded environments, ranging from discrete DES complexes to bulk-like acid clusters.

Low-frequency bands at 537–532.5 cm^–1^ and 336–331.5 cm^–1^ are attributed to the skeletal deformation modes of the MSA molecule. Particularly, the lattice-like vibrations observed in the 75–70.5 cm^–1^ range become weaker as the MSA concentration increases. Unlike FTIR, which is often featureless in this range, these Raman lattice modes provide exclusive evidence for the complete disruption of the HACl crystal lattice, supporting the transition into a stabilized and highly fluidic, yet structured, protic ionic medium.

Raman results also correlate with the FTIR data, and taken together, a portrait emerges of a structural transformation: when the ratio is 1:4, hydrogen bonding is favored, and at a ratio of 1:8, a highly acidic MSA-based ion network is formed. This further supports the tunable nature of the HACl-MSA mixture, in which the molecular environment is effectively tailored for specific applications in nonaqueous extractive metal processes.

### 3.4 NMR Spectroscopy Analysis

The ^1^H NMR spectroscopy provides critical insights into the molecular interactions and structural dynamics of DESs formed by HACl and MSA across molar ratios from 1:4 to 1:8. Figures 4 and S4 show the ^1^H NMR spectra of DESs and their constituent compounds as stacked and individually separated spectra, respectively. Table 2 summarizes the peak positions and assignments, detailing both absolute chemical shifts (δ, ppm) and corresponding relative chemical shift variations (Δδ, ppm) calculated relative to pure starting components.

**Figure 4:**
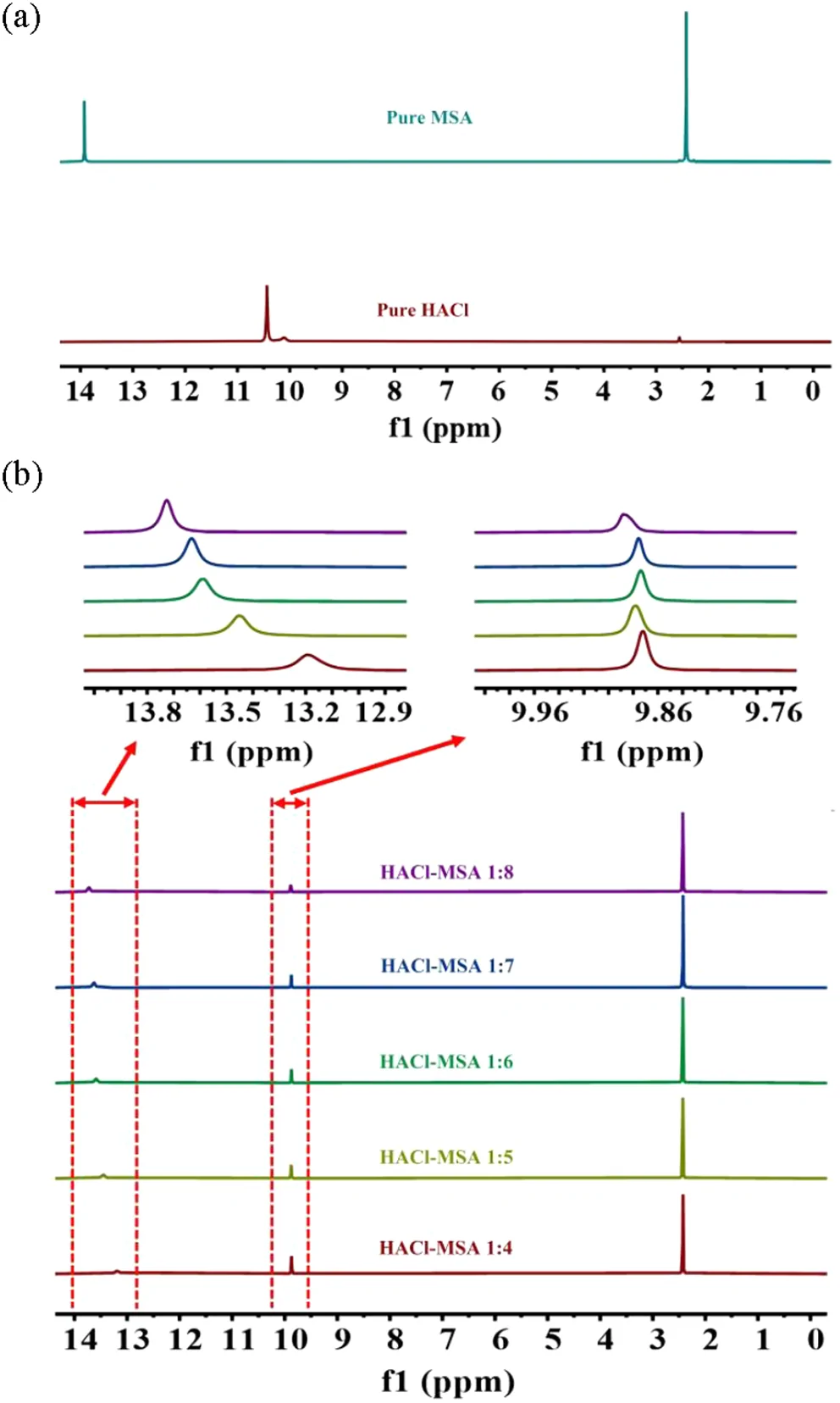
^1^H NMR spectra of HACl-MSA in various molar ratios (a, b).

**Table 2:** Observed ^1^H NMR Chemical Shifts (δ, ppm) and Relative Chemical Shift Variations (Δ*δ*, ppm) for Pure Components and HACl–MSA DES Systems at Varying Molar Ratios and Room Temperature

sample	acid proton pool (δ, ppm)	Δ*δ*_acid_ relative to pure MSA (ppm)^a^	[NH_3_OH]^+^ core (δ, ppm)	Δ*δ* relative to HACl (ppm)^b^	–CH_3_ (δ, ppm)	Δ*δ*_CH_3__ (ppm)^c^
pure HACl	—	—	10.44/10.11	—	—	—
pure MSA	13.92	—	—	—	2.42	—
HACl–MSA (1:4)	13.19	–0.73	9.87	–0.57/–0.24	2.43	+0.01
HACl–MSA (1:5)	13.45	–0.47	9.88	–0.56/–0.23	2.43	+0.01
HACl–MSA (1:6)	13.59	–0.33	9.87	–0.57/–0.24	2.43	+0.01
HACl–MSA (1:7)	13.63	–0.29	9.88	–0.56/–0.23	2.42	0.00
HACl–MSA (1:8)	13.73	–0.19	9.88	–0.56/–0.23	2.42	0.00

a Calculated as δ_DES_ – δ_MSA_ (13.92 ppm).

b Calculated relative to neat HACl peaks (10.44 and 10.11 ppm).

c Calculated as δ_CH_3_–DES_ – δ_CH_3_–MSA_ (2.42 ppm).

As illustrated in Figure 4a and presented in Table 2, pure MSA exhibits two distinct signals: a broad peak at 13.92 ppm, assigned to the sulfonic acid proton (−SO_3_H), which signifies a highly deshielded, free-acid environment typical for a strong acid with intermolecular hydrogen bonding. The sharp singlet at 2.42 ppm corresponds to the methyl (−CH_3_) protons, deshielded by the electron-withdrawing sulfonate group, and serves as a highly stable structural reference. The spectrum of pure HACl reveals two characteristic exchangeable proton signals: a sharp resonance at 10.44 ppm (NH_3_^+^) and a broader signal at 10.11s ppm (OH). The five-peak cluster centered at 2.56 ppm is the residual signal of the DMSO-*d*_6_ solvent.

Upon DES formation (Figure 4b), the HACl-MSA system collapses into three primary signals across all ratios from 1:4 to 1:8, indicating the formation of the homogeneous and single-phase DES. The disappearance of the individual HACl peaks and the significant shift of the MSA acid proton support the integration of all protic species into the eutectic matrix. The −CH_3_ signal remains exceptionally stable at 2.42–2.43 ppm with a negligible shift variation (Δδ_CH_3__ ≤ + 0.01 ppm relative to pure MSA), matching pure MSA at higher ratios. This consistency indicates that the alkyl backbone remains nonlabile and uninvolved in the exchange dynamics, providing a reliable internal reference throughout the composition range.

The DESs exhibit a single-phase structure with rapid internal proton exchange, enabling tunable acidity and a transition to a more ionic environment at higher MSA ratios. As presented in Table 2, the spectra show signals for the 1:4 ratio at 13.19 and 9.87 ppm, shifting to 13.73 and 9.88 ppm for the 1:8 ratio. The reduction to three signals across all ratios is indicative of a fast chemical exchange regime within specific acidic and hydroxyl pools. NMR analysis reveals two distinct exchangeable proton environments coexisting in the liquid phase.

The first is a consistent low-field peak at 9.87–9.88 ppm, which appears significantly shielded compared to the original HACl signals (Δδ = −0.56 to −0.57 ppm relative to neat 10.44 ppm, and Δδ = −0.23 to −0.24 ppm relative to neat 10.11 ppm). This resonance represents the stable hydroxylammonium cation ([NH_3_OH]^+^) core. Its narrow shift range and near-zero shift change across the DES compositions (Δδ < 0.01 ppm between 1:4 and 1:8) suggest that these protons are involved in localized, saturated hydrogen-bonding interactions with sulfonate anions, establishing a robust structural unit regardless of excess MSA.

The second environment is a dynamic acid proton pool at 13.19–13.73 ppm, representing an average of the exchangeable protons of the MSA and the acidic network. To distinguish pure component baselines from trends across the DES series, shifts are evaluated both relative to neat components and across the compositional range. At a 1:4 ratio, this signal exhibits an initial upfield shift of Δδ = −0.73 ppm relative to pure MSA (13.92 ppm), driven by initial network disruption upon cation coordination. As the MSA ratio increases from 1:4 to 1:8, these peaks shift progressively downfield across the series (Δδ_series_ *=* +0.54 moving from 13.19 ppm at 1:4 to 13.73 ppm at 1:8), reducing the relative upfield deviation from pure MSA (from Δδ = −0.73 ppm at 1:4 to Δδ = −0.19 ppm at 1:8). This trend reflects the transition toward a strongly Brønsted-acidic regime, where increased MSA concentration enhances the deshielding of the proton pool through an intensified hydrogen-bonding network.

The ^1^H NMR results further validate the observations of the vibrational spectra, reflecting structural transformation on the molecular level. The acid proton pool shifts downfield and sharpens (δ *=* 13.19 to 13.73 ppm across the series), consistent with the red shift of the symmetric S=O stretch in the FTIR (1145.95 to 1131.68 cm^–1^) and the broadening of the S–OH band in Raman (904 cm^–1^). Together, these techniques describe a structural evolution where the MSA-MSA hydrogen-bonded network dominates as the ratio increases, facilitating faster proton dynamics and higher operational Brønsted acidity. While FTIR and NMR indicate a trend toward shorter, more acidic hydrogen bonds, the Raman broadening highlights the environmental heterogeneity and fluidity of this bulk acid network. This transition facilitates enhanced proton dynamics and a marked increase in bulk acidity. Furthermore, the stability of the [NH_3_OH]^+^ core in NMR mirrors the persistence of the N–O stretching band at 1158 cm^–1^ in the Raman spectra, suggesting that the cation remains a stable structural anchor even in the strongly acidic regime.

To evaluate the nature of intermolecular interactions relative to complete proton transfer, the spectroscopic data were carefully examined. The retention of the characteristic S–OH stretching vibration (∼904–920 cm^–1^) in both FTIR and Raman spectra indicates that permanent, complete deprotonation of MSA does not occur in the bulk phase. Likewise, the ^1^H NMR spectra show a single, exchange-broadened downfield signal rather than separate signals for distinct protonated and deprotonated species, reflecting rapid chemical exchange on the NMR time scale. While these steady-state measurements strongly support the presence of a dynamic, highly polarizable hydrogen-bonded network, they cannot conclusively exclude transient or partial proton-transfer states that may operate on ultrafast time scales.

To further evaluate the uniqueness of the HACl–MSA system, its NMR behavior was compared with previously reported protic and strongly acidic DES systems in the literature, such as ammonium acetate–methanesulfonic acid (AAc–MSA)^25^ and urea–methanesulfonic acid (Urea–MSA).^22^ In all MSA-based systems, the methyl (−CH_3_) singlet remains exceptionally invariant at approximately 2.42–2.43 ppm, highlighting the universal nonparticipatory nature of the MSA aliphatic backbone. However, the exchange dynamics of the protic species reveal distinct structural behaviors across different HBAs. In AAc–MSA systems, strong basic anion protonation leads to a complete collapse of all protic species into a single, fast-exchanging dynamic proton pool across all ratios. In contrast, the HACl–MSA system preserves two distinct exchangeable proton environments ([NH_3_OH]^+^ at 9.87–9.88 ppm and the dynamic acid pool at 13.19–13.73 ppm). This dual-environment persistence indicates that the hydroxylammonium cation forms a far more rigid, localized structural core than acetate-based systems. In Urea–MSA systems, Brønsted acidity is driven by carbonyl O-protonation and an evolving amide proton exchange network. Conversely, HACl–MSA utilizes a chloride-mediated hydrogen-bonding manifold (S–OH···Cl^–^) that sustains rapid acid pool proton mobility while isolating the cationic anchor. This comparative analysis highlights that while MSA ratio expansion universally drives downfield deshielding toward a strongly acidic regime, the HACl–MSA system uniquely balances high dynamic acid fluidity with a structurally stable cation core.

Collectively, this multispectroscopic approach supports the interpretation that the HACl–MSA system is a highly tunable, strongly Brønsted-acidic solvent platform. By modulating the molar ratio, the molecular environment can be precisely adjusted to optimize the selective leaching and reductive recovery of metals in advanced metallurgical applications. The combined results of the above-mentioned spectroscopic analyses highlight the versatility and superior acidity of the HACl–MSA system as a highly acidic system that can be varied by the ratio for selective leaching and recovery of metals for metallurgical applications.

### 3.5 Hammett Acidity Function (*H*_0_) Analysis

The Brønsted acidity of the HACl–MSA DES series was evaluated using the Hammett acidity function (*H*_0_) with 4-nitroaniline for aqueous mixtures and 2,4-dinitroaniline for pure DES formulations. Evaluating *H*_0_ in neat ionic solvents introduces matrix effects such as local dielectric alterations, solvatochromic baseline shifts, and potential variations in indicator *pK*_a_ and molar extinction coefficients relative to standard reference media. Consequently, the *H*_0_ values reported herein represent operational, indicator-dependent acidity measurements. Nevertheless, when evaluated under standardized conditions across the composition series, these operational metrics provide a consistent basis for comparing relative proton availability. The acidity results, graphically shown in Figure 5 and summarized in Tables 3 and Table 4, reveal the exceptional acidity of these DESs, particularly as the MSA content increases toward the 1:8 molar ratio.

**Figure 5:**
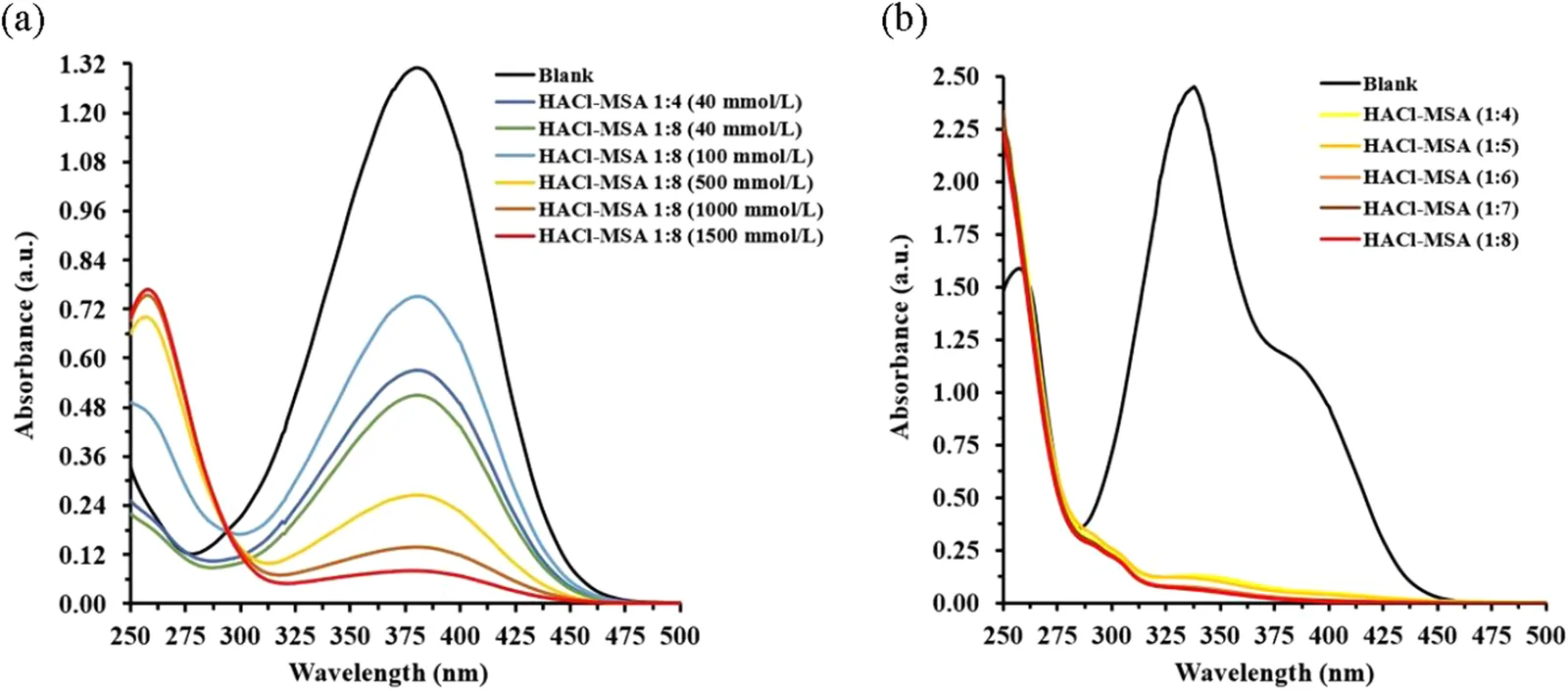
UV–vis absorbance spectra of (a) 4-nitroaniline at a concentration of 0.1 mmol/L in water for HACl-MSA DESs at molar ratios of 1:4 and 1:8 (40.0 mmol/L) and 1:8 at various concentrations (up to 1500 mmol/L) at room temperature, and (b) 2,4-dinitroaniline at a concentration of approximately 1.7 × 10^–1^ mmol/L in ethanol for pure HACl-MSA DESs at 1:4 and 1:8 ratios.

**Table 3:** Hammett Acidity Function (*H*_0_) Values for Aqueous HACl-MSA DESs at Concentrations of 40–1500 mmol/L and at Room Temperature, Measured with 4-Nitroaniline at a Concentration of 0.1 mmol/L, Compared with Literature Data^a^

	*A*_max_	[*I*] (%)	[*IH* ^+^] (%)	*H*_0_	RMSE^b^
blank	1.309	100	0		0.0003
HACl-MSA (1:4) 40 mmol/L	0.572	0.437	0.563	0.879	0.0036
HACl-MSA (1:8) 40 mmol/L	0.510	0.389	0.611	0.794	0.0050
HACl-MSA (1:8) 100 mmol/L	0.750	0.573	0.427	1.118	0.0005
HACl-MSA (1:8) 500 mmol/L	0.266	0.203	0.797	0.396	0.0006
HACl-MSA (1:8) 1000 mmol/L	0.139	0.106	0.894	0.066	0.0002
HACl-MSA (1:8) 1500 mmol/L	0.079	0.061	0.939	–0.200	0.0002
[Mimps][H_2_SO_4_][Bibr ref34] ^c^	0.172	55	45	1.07	
[Mimps][HCl][Bibr ref34] ^c^	0.186	59	41	1.15	
[Mimps][Tfs][Bibr ref34] ^c^	0.208	66	34	1.28	
[Bimps][H_2_SO_4_][Bibr ref34] ^c^	0.186	59	41	1.14	
[Bimps][HCl][Bibr ref34] ^c^	0.197	63	37	1.22	
[Oimps][H_2_SO_4_][Bibr ref34] ^c^	0.197	62	38	1.21	
[Oimps][HCl][Bibr ref34] ^c^	0.223	71	29	1.38	
[Choline][HSO_4_][Bibr ref35] ^d^	1.19	81.5	18.5	1.63	
[Choline][H_2_PO_4_][Bibr ref35] ^d^	1.27	86.9	13.1	1.81	
[DMEA][HSO_4_][Bibr ref35] ^d^	0.43	29.5	70.5	0.61	
[DMEA][H_2_PO_4_][Bibr ref35] ^d^	1.20	82.2	17.8	1.65	
[PSMim][HSO_4_][Bibr ref36] ^e^	1.393	59.2	40.8	1.15	
[PSMim][H_2_PO_4_][Bibr ref36] ^e^	1.912	81.3	18.7	1.63	
[PSPy][HSO_4_][Bibr ref36] ^e^	1.537	65.3	34.7	1.26	
[PSPy][H_2_PO_4_][Bibr ref36] ^e^	2.038	86.6	13.4	1.80	
[PSTma][HSO_4_][Bibr ref36] ^e^	1.689	71.8	28.2	1.40	
[PSPy][HSO_4_][Bibr ref36] ^e^	1.537	65.3	34.7	1.26	
[C_3_SO_3_Hmim][HSO_4_][Bibr ref37] ^c^	0.64	67	33	1.30	
[C_3_SO_3_Hmim][Cl][Bibr ref37] ^c^	0.80	84	16	1.71	
[DMEA][Cl][Bibr ref38] ^d^	2.467	80.7	19.3	1.61	
[DMEA][OAc][Bibr ref38] ^d^	1.972	64.5	35.5	1.25	
[DMEA][HSO_4_][Bibr ref38] ^d^	0.816	26.7	73.3	0.55	
ChCl-AP (1:2)^39^ ^f^	0.998	94.6	5.4	2.23	
ChCl-MCA (1:2)^39^ ^f^	0.790	74.9	25.1	1.46	
ChCl-TCA (1:2)^39^ ^f^	0.508	48.2	51.8	0.96	

a The standard uncertainty is *u*(*H*_0_) = 0.02.

b The RMSE was calculated across three independent replicates.

c The concentrations of IL and 4-nitroaniline were 50.0 mmol/L and 3 mg/L in water, respectively.

d The concentrations of IL and 4-nitroaniline were 20.0 mmol/L and 100 mg/L in water, respectively.

e The concentrations of IL and 4-nitroaniline were 5.0 mmol/L and 10 mg/L in dichloromethane, respectively.

f The concentrations of DES and 4-nitroaniline were 40.0 mmol/L and 10 mg/L in water, respectively.

**Table 4:** Hammett Acidity Function (*H*_0_) Values of Pure HACl-MSA DESs at Room Temperature, Measured with 2,4-Dinitroaniline at a Concentration of 1.7 × 10^–1^ mmol/L in Ethanol^a^

sample	*A*_max_	[*I*] (%)	[*IH* ^+^] (%)	*H*_0_	RMSE^b^
blank	2.448	100	0		0.0022
HACl-MSA 1–4	0.130	5.3	94.7	–5.782	0.0055
HACl-MSA 1–5	0.117	4.8	95.2	–5.828	0.0025
HACl-MSA 1–6	0.074	3.0	97.0	–6.037	0.0018
HACl-MSA 1–7	0.067	2.7	97.3	–6.080	0.0017
HACl-MSA 1–8	0.062	2.5	97.5	–6.116	0.0012

a The standard uncertainty is *u*(*H*_0_) = 0.02.

b RMSE was calculated across three independent replicates.

Figure 5a depicts the UV–vis spectra of 4-nitroaniline in aqueous solutions. According to data in Table 3, the *H*_0_ values of the aqueous solution range from 0.879 for the ratio of 1:4 at 40 mmol/L to −0.200 for the ratio of 1:8 at 1500 mmol/L, depicting an expected trend, where the acidity increases with increased MSA concentration, along with the total concentration. The HACl-MSA (1:8)/1500 mmol/L solution is a strong acid, as indicated by an *H*_0_ = −0.200, and is accompanied by a high protonation state of 93.9% in 4-nitroaniline. On the other hand, the HACl-MSA (1:4)/40 mmol/L has an *H*_0_ = 0.879, which illustrates relatively lower acidity, thereby establishing lower protonating ability in HACl compared to MSA. This concentration-dependent trend correlates with the spectroscopic observations: as MSA concentration rises, the ″acidic proton pool″ identified in NMR becomes more deshielded and active. When compared to literature values for other acidic systems such as ionic liquids (ILs) ([DMEA]­[HSO_4_] with *H*_0_ = 0.55) and other DESs (ChCl-AP (1:2) with *H*_0_ = 2.23), the HACl-MSA (1:8) DES at 1500 mmol/L stands out as one of the most potent acidic media reported.

For pure HACl-MSA DESs (Table 4), measured in ethanol with 2,4-dinitroaniline, the *H*_0_ values range from −5.782 (1:4 ratio) to −6.116 (1:8 ratio), indicating highly acidic behavior. Figure 5b depicts the UV–vis spectra of 2,4-dinitroaniline in pure DESs. With increasing MSA ratio, acidity increases, and the protonated indicator concentration, [*IH* ^+^], changes from 94.7 to 97.5%, indicating the predominant role of MSA as a proton donor. The occurrence of such a low value of *H*_0_ indicates the potential application of HACl-MSA-based DESs in highly acidic processes like metal extraction. The difference between the *H*_0_ values in aqueous solutions of DESs (Table 3) and pure DESs (Table 4) highlights the effect of water as a solvent in reducing acidity because of its ability to interact with protons.

This high acidity arises from the molecular transitions described in the previous sections. When the system reaches the 1:8 ratio, a dominant MSA–MSA hydrogen-bonded network is formed, as supported by the NMR downfield shift to 13.63 ppm, the red-shifting of the symmetric S=O stretch in FTIR (1145 to 1131 cm^–1^), and Raman broadening of the S–OH modes. Such a network provides an extremely favorable environment for proton donation. Unlike water, which mollifies acidity through strong solvation, the DES matrix keeps these “pool” protons highly labile and ready to transfer. The large difference between the aqueous solution (*H*_0_ ≈ −0.2) and pure DES (*H*_0_ ≈ −6.1) strongly indicates the significant effect of solvent interactions. In the latter, where there is absolutely no water, there will be no leveling effect, hence allowing the full strength of the acidic properties of the MSA-dominated matrix to become evident.

The HACl-MSA DESs provide a level of tunability in acidity, which can be varied by adjusting molar ratios and concentration. Such variability in acidity is a huge advantage in metallurgical applications. Although the molar ratio of 1:8 possesses the highest protonating ability, its effectiveness also depends on the number of available coordination sites in this solvent. A higher concentration of salt, for instance, at a HACl/MSA (1:4), creates a far superior coordination environment because of its greater concentration of hydroxylammonium ion [NH_3_OH]^+^ and chloride ion Cl^–^ species. It is evident from spectroscopic analyses that in these lower molar ratios, these components are not as deeply sequestered within the MSA-MSA matrix. Instead, they act as a ligand and a stabilizer. This is a good combination since this environment is highly acidic and has active sites for solvating and stabilizing a complex. Thus, HACl-MSA is not a proton source; it is a highly tunable coordination platform wherein the extreme bulk acidity is balanced by the required coordination-driven stability for advanced metal extraction through ratio tuning. Therefore, the HACl-MSA system is more than just a proton source; it is a highly tunable coordination platform where the ratio can be precisely adjusted to optimize the trade-off between extreme bulk acidity and coordination-driven stability. These tunable properties position HACl-MSA DESs as a versatile and sustainable alternative for selective metal recovery.

While highly acidic DESs hold significant potential for sustainable hydrometallurgical applications, establishing their fundamental physical chemistry, dynamic proton networks, and quantitative acidity metrics is an essential prerequisite for rational process design. Herein, the structural and acidity characterization of the HACl–MSA DES system serves as a foundational platform for upcoming extractive and metallurgical evaluations across different types of ores.

### 3.6 Computational Results and Analysis

For each stoichiometric ratio, the thermodynamically favored DES configuration was identified by evaluating the |Δ*E*_int_|. Additional optimized structural geometries for HACl–MSA (1:4) and (1:5) are provided in Figure S5. The electronic structure and stability of the HACl-MSA DESs were evaluated through ESP maps and FMO analysis. These results, summarized in Table 5, elucidate the fundamental interactions between the HACl and MSA components. The ESP maps (Figure 6, left side) illustrate the distribution of nucleophilic (electron-rich) and electrophilic (electron-deficient) regions across the molecules. For pure HACl and MSA, the ESP maps identify the specific sites available for hydrogen bonding, with the sulfonic acid proton of MSA serving as a primary site for interaction. Upon complexation at 1:4 and 1:5 molar ratios, the ESP maps show a redistribution of electron density, confirming the formation of a robust, unified hydrogen-bonded network.

**Table 5:** Electronic Parameters of the Isolated HACl, MSA, and DESs

system	|Δ*E*_int_| (eV)	E_HOMO_ (eV)	E_LUMO_ (eV)	*E*_gap_ (eV)	EA = −E_LUMO_	IP = −E_HOMO_	μ (eV)	η (eV)	*S* (eV^–1^)	ω (eV)	χ (eV)
HACl		–8.331	–0.775	7.556	0.775	8.331	–4.553	3.778	0.132	2.743	4.553
MSA		–8.727	–0.776	7.951	0.776	8.727	–4.751	3.975	0.126	2.839	4.751
HACl-MSA (1–4)	3.595	–8.168	–0.844	7.324	0.844	8.168	–4.506	3.662	0.137	2.772	4.506
HACl-MSA (1–5)	4.280	–8.156	–0.715	7.441	0.715	8.156	–4.436	3.721	0.134	2.644	4.436

**Figure 6:**
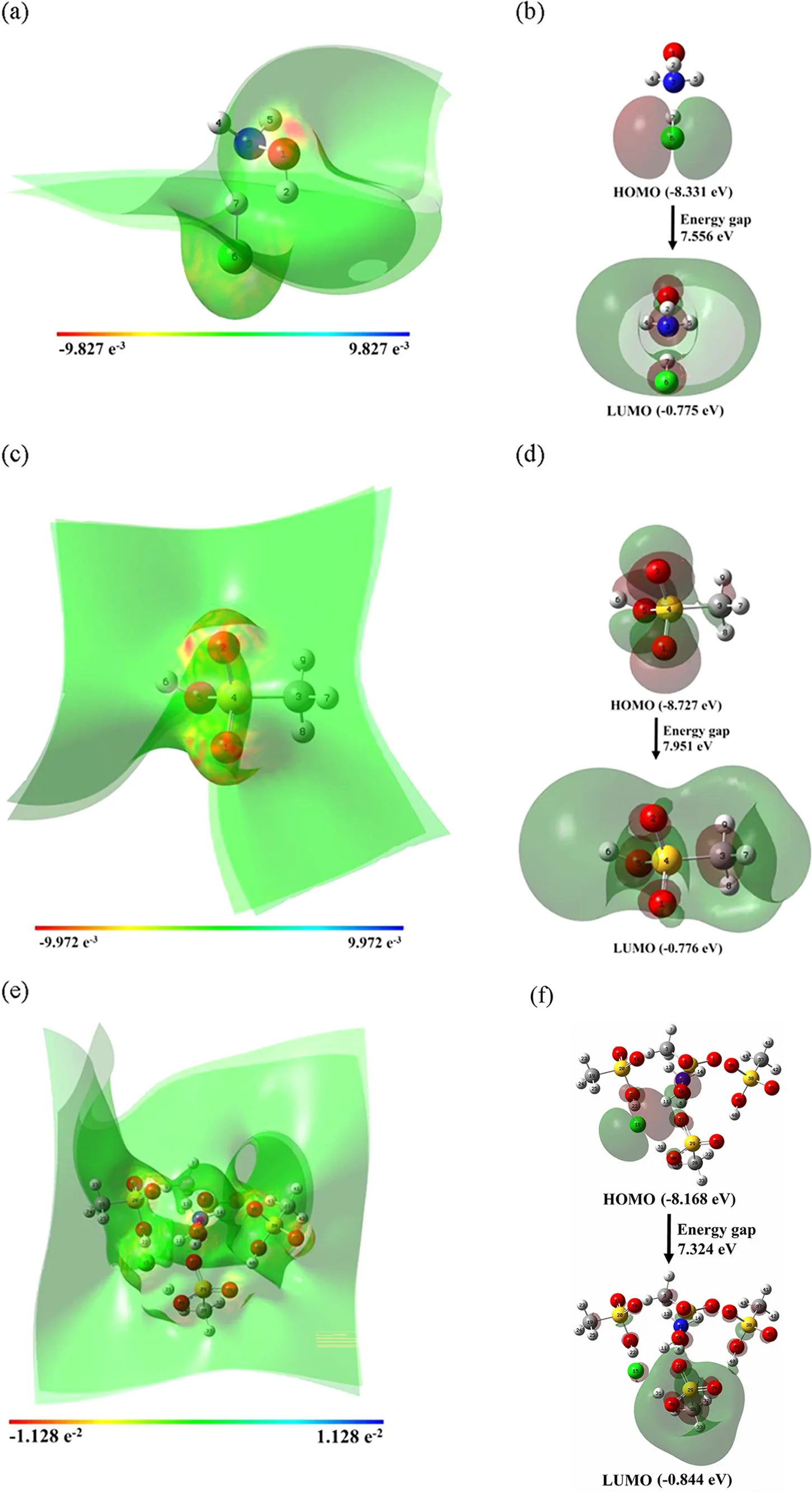

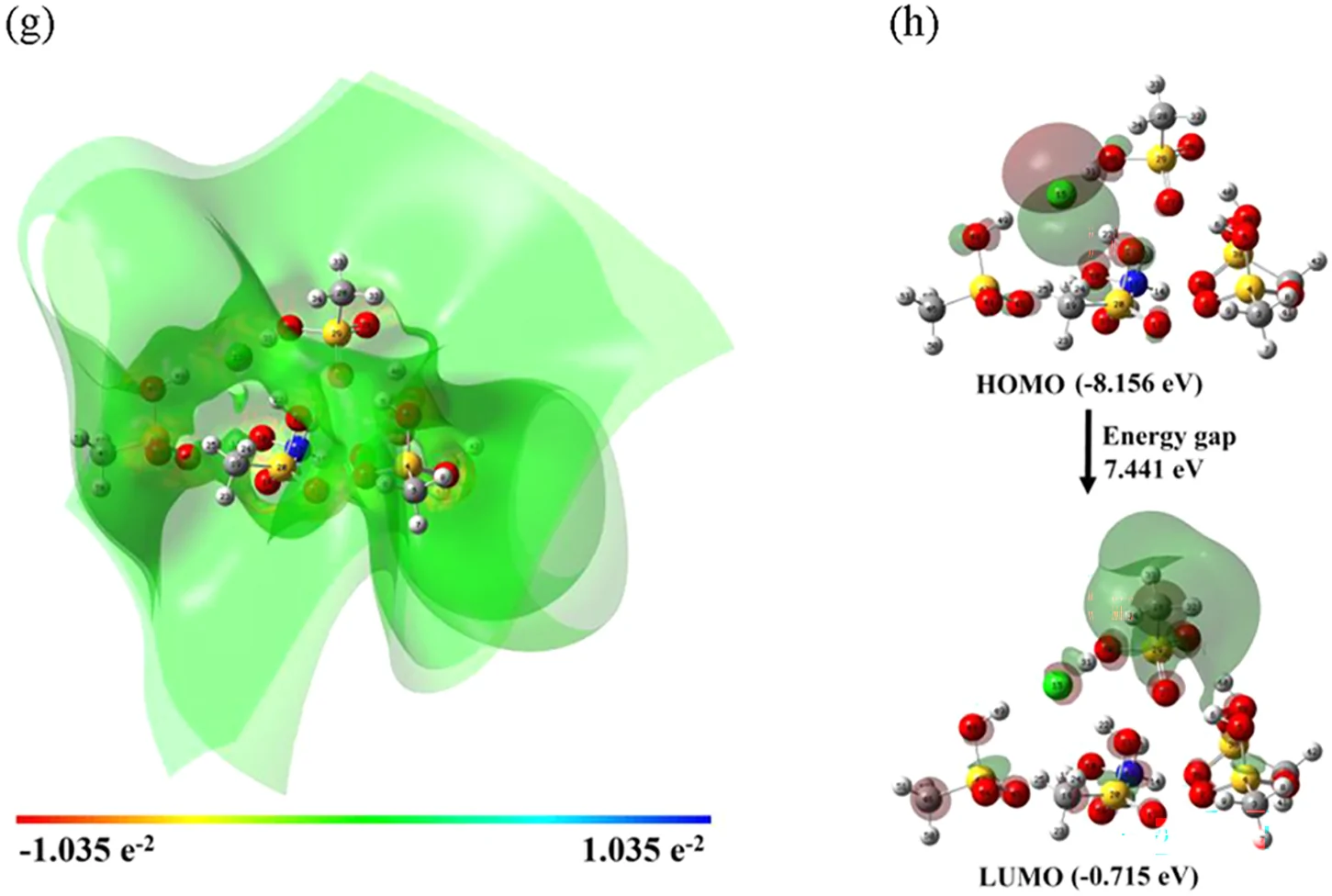
ESP map and HOMO–LUMO distributions: (a, b) HACl, (c, d) MSA, (e, f) HACl-MSA (1:4), and (g, h) HACl-MSA (1:5). Atom color coding: Cl (green), H (light gray), C (dark gray), S (yellow), O (red), and N (blue).

For pure HACl, the ESP map (Figure 6a) reveals a specific distribution of electron density following geometry optimization. A high concentration of negative potential, indicated by orange regions, is localized around the chloride anion (Cl^–^), specifically where it forms a close Cl···H interaction with a hydrogen atom from the cation. Additionally, a slight red color is observed on one side of the oxygen atom, identifying it as a secondary nucleophilic site within the hydroxylammonium ([NH_3_OH]^+^) cation. Conversely, the nitrogen atom appears electronically neutral or ″transparent″ in the ESP map, showing no distinct color concentration. In contrast, the ESP map for pure MSA (Figure 6c) reveals a distinct electronic asymmetry characterized by the localization of nucleophilic potential specifically around the two sulfonyl oxygen atoms (O=S=O), as indicated by red/orange contours. These regions identify the sulfonyl oxygens as the primary HBA sites, while the hydroxyl (−OH) group remains electronically moderated, facilitating its role as the primary HBD.

Upon complexation at 1:4 and 1:5 molar ratios, the ESP maps demonstrate a significant redistribution of these localized potentials as the nucleophilic density previously restricted to the Cl^–^ and O=S=O sites is integrated into a broader, shared potential surface. This transition confirms the formation of a robust, unified hydrogen-bonded network where the individual electronic identities of the salt and acid are subsumed into a cohesive eutectic matrix. The thermodynamic stability of this network is confirmed by the |Δ*E*_int_|, which increases from 3.595 eV for the 1:4 ratio to 4.280 eV for the 1:5 ratio. This significant rise in |Δ*E*_int_| indicates that the system becomes increasingly stable as the MSA-MSA network expands, supporting the experimental observation of a more extensively integrated matrix at higher ratios.

The FMO analysis (Figure 6b,d,f,h, right side) and global reactivity descriptors offer valuable insights into the electronic reactivity of these systems for potential metal extraction applications. For pure HACl, the HOMO is concentrated around the chloride anion, while the LUMO is localized on the hydroxylammonium cation. In pure MSA, the HOMO is distributed over the sulfonate oxygen atoms. The calculated energy gap (*E*_gap_) for the 1:4 and 1:5 complexes (7.324 and 7.441 eV, respectively) is significantly lower than that of pure MSA (7.951 eV), signaling a marked increase in chemical reactivity upon eutectic formation. Notably, the 1:4 ratio exhibits the lowest energy gap and the highest global softness (*S* = 0.137 eV^–1^), alongside a higher electrophilicity index (ω = 2.772 eV). These parameters indicate that the 1:4 ratio is more electronically polarizable and reactive toward external solutes compared to the 1:5 ratio. Furthermore, the 1:4 ratio possesses a more negative chemical potential (μ = −4.506 eV) compared to the 1:5 ratio (μ = −4.436 eV), suggesting a stronger driving force for electronic exchange. While the 1:5 ratio is more internally stable due to its higher |Δ*E*_int_|, the 1:4 ratio provides a highly polarizable electronic environment; its higher softness and chemical potential suggest a favorable theoretical framework for charge transfer and metal ion coordination during leaching. However, while these DFT findings highlight the 1:4 ratio as a promising theoretical candidate for metal coordination, definitive conclusions regarding its operational suitability for metallurgical processes require future experimental evaluation of extraction yields, leaching kinetics, viscosity, and metal selectivity.

Beyond global electronic descriptors, the theoretical suitability of the 1:4 molar composition for potential extractive metallurgical applications is supported by a balance of ligand stoichiometry, structural mobility, and acidity. Stoichiometrically, the 1:4 ratio provides the highest volumetric concentration of active [NH_3_OH]^+^ reductants and Cl^–^ coordinating species (20 mol % HACl compared to 11.1 mol % at 1:8). Furthermore, DFT interaction energy calculations reveal a lower cluster binding energy for the 1:4 system (|Δ*E*_int_| = 3.595 eV) relative to 1:5 (4.280 eV), indicating a less rigid supramolecular matrix that enhances molecular mobility and mass transport during leaching. While higher MSA proportions incrementally increase Brønsted acidity (*H*_0_ = −6.116 for 1:8 vs −5.782 for 1:4), the 1:4 system already provides sufficient acidity for matrix dissolution without compromising the concentration of essential reducing and complexing agents. Nevertheless, these theoretical and stoichiometric features serve to identify 1:4 as a candidate for future investigation rather than demonstrating superior macroscopic extraction performance, selectivity, stability, or recyclability.

### 3.7 Quantitative Spectroscopic–Acidity Correlations

To establish a quantitative understanding of the structural framework governing Brønsted acidity in the HACl–MSA DES series, experimental spectroscopic parameters, Hammett acidity measurements, and complementary DFT-derived interaction energies were systematically examined. The experimental correlations were established across the HACl–MSA composition range (1:4–1:8) (Figure 7), while the DFT calculations for the representative 1:4 and 1:5 DESs provide complementary quantum chemical support for the observed trends.

**Figure 7:**
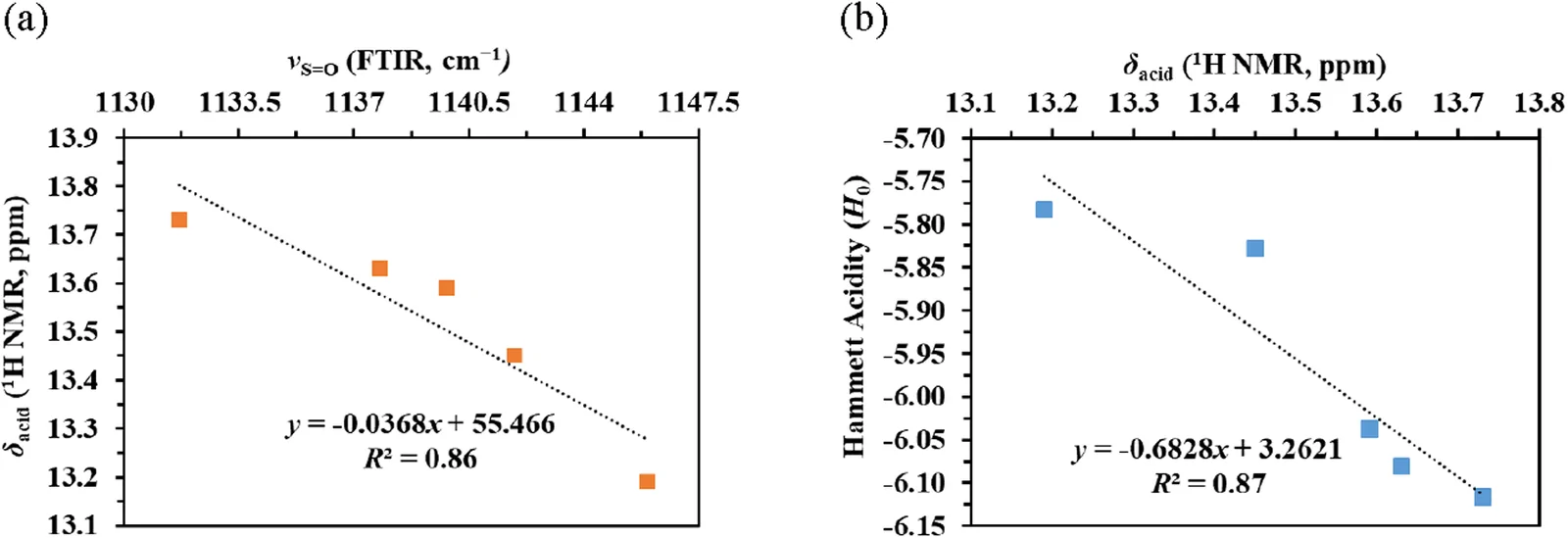
Linear correlations between (a) FTIR stretching frequency (ν_S=O_) and ^1^H NMR chemical shift (δ_acid_), and (b) ^1^H NMR chemical shift (δ_acid_) and Hammett acidity (*H*_0_).

Linear regression analysis reveals an approximately linear relationship (*R* ^2^ = 0.86, *n* = 5) between the symmetric S=O stretching frequency (ν_S=O_) obtained from FTIR and the ^1^H NMR chemical shift of the acidic proton resonance (δ_acid_), as displayed in Figure 7a

12 $$\delta_{\mathrm{acid}}=-0.0368\nu_{S=O}+55.466$$

As the HBD content increases from 1:4 to 1:8, the progressive red shift in ν_S=O_ (Δ*ν* = −14.27 cm^–1^, moving from 1145.95 to 1131.68 cm^–1^) is accompanied by a continuous downfield movement of δ_acid_ (Δδ = +0.54 ppm, moving from 13.19 to 13.73 ppm). This trend indicates that enhanced participation of sulfonyl oxygens in hydrogen bonding (S=O···H–O) is accompanied by increased proton deshielding, resulting in the observed downfield ^1^H NMR shifts.

A comparison between the acidic proton chemical shift and the Hammett acidity function reveals an approximately linear relationship (*R* ^2^ = 0.87, *n* = 5) between δ_acid_ and *H*_0_, as displayed in Figure 7b

13 $$H_{0}=-0.683\delta_{\mathrm{acid}}+3.26$$

Across the evaluated composition bounds, progressive downfield shifts in δ_acid_ correspond to lower *H*_0_ values (decreasing from −5.782 at 1:4 to −6.116 at 1:8). Because these regressions are based on five composition points, they must be interpreted strictly as exploratory empirical trends within the investigated stoichiometric window rather than generalized predictive models. Furthermore, it is important to acknowledge that component stoichiometry (composition) serves as a common underlying variable that simultaneously drives both the microscopic proton deshielding and macroscopic acidity.

Finally, the DFT calculations provide complementary quantum chemical support for the experimentally observed correlations. The magnitude of the **|**Δ*E*_in**t**_ **|** increases from 3.595 eV for HACl–MSA (1:4) to 4.280 eV for HACl–MSA (1:5), indicating stronger intermolecular interactions and enhanced thermodynamic stabilization of the hydrogen-bonding network upon complexation. This increased stabilization is consistent with the progressive FTIR red shifts, the increased proton deshielding observed by ^1^H NMR, and more negative Hammett acidity values. However, because DFT calculations were conducted on two discrete compositions (1:4 and 1:5), these computational results serve to illustrate local bonding interactions rather than validating or extrapolating a continuous trend across the complete 1:4–1:8 series. Together, these complementary experimental and computational results support the proposed relationship between hydrogen-bond strengthening, the local proton environment, and macroscopic Brønsted acidity in the HACl–MSA DES system.

## 4 Conclusion

This research has successfully introduced a new deep eutectic solvent (DES) composed of hydroxylamine hydrochloride (HACl) and methanesulfonic acid (MSA), defining a complete structure–property correlation for the 1:4 to 1:8 HACl/MSA ratio. Thermal analysis via DSC revealed composition-dependent phase behavior, transitioning from an amorphous glass-forming liquid at a 1:4 ratio (*T*_g_ = −88.51 °C) to congruent melting at 1:5 (*T*_m_ = +5.43 °C) and invariant eutectic solidus–liquidus regimes across 1:6–1:8 formulations. FTIR, Raman, and ^1^H NMR analyses collectively provided definitive proof for a protic single-phase DES with a strong hydrogen-bonding network and a shared electronic potential for the HACl/MSA DESs. The ^1^Hs NMR analyses confirmed the presence of two separate regions of protons within the study: a stable, shielded hydroxylammonium core, [NH_3_OH]^+^, at 9.87–9.88 ppm, and a dynamic, downfield-shifting acid pool for the MSA’s acid protons at 13.19–13.63 ppm. This shifting resonance directly reflects a tunable macroscopic acidity where the expanding MSA-MSA network facilitates rapid proton exchange. Quantitative Hammett acidity function (*H*_0_) measurements confirmed that the pure DESs exhibit significant, tunable Brønsted acidity, reaching *H*_0_= −6.116 at the 1:8 ratio. Moreover, even when diluted, this system is still strongly acidic and controllable.

Density functional theory (DFT) calculations and global reactivity indices provided important molecular-level insights that further support these results. ESP maps confirmed an electronic asymmetry in MSA, where the sulfonyl oxygens (O=S=O) act as primary acceptors and the hydroxyl group functions as the primary donor. Although the 1:5 ratio showed a higher thermodynamic stability (**|**Δ*E*_int_ **|** = 4.280 eV), the 1:4 ratio was identified as the most reactive and polarizable medium, characterized by the lowest *E*_gap_ (7.324 eV) and the highest global softness (*S* = 0.137 eV^–1^). Furthermore, the 1:4 ratio demonstrated a more negative chemical potential (μ = −4.506 eV) compared to the 1:5 ratio (μ = −4.436 eV), indicating a greater driving force for electronic exchange. These computational analyses show that the HACl-MSA 1:4 ratio has a more favorable electronic environment for metallurgical applications. The lower *E*_gap_ and higher global softness values calculated for the 1:4 ratio suggest a highly polarizable medium that may offer a favorable electronic environment for solute interactions compared with HBD-rich ratios. However, while quantum chemical reactivity indices highlight the 1:4 formulation as a promising theoretical candidate, definitive conclusions regarding its suitability for metallurgical applications require comprehensive experimental studies on leaching kinetics, metal selectivity, viscosity, and solvent stability.

In summary, the HACl-MSA DES is a novel, bifunctional platform where operational Brønsted acidity and electronic reactivity can be systematically tuned via molar composition of the eutectic components. This dual tunability, combined with the inherent reducing capacity of the hydroxylamine moiety, identifies HACl-MSA DESs as promising candidate solvents for future hydrometallurgical investigations. However, claims regarding operational metal extraction selectivity, leaching efficiency, solvent recyclability, degradation stability, and overall process sustainability represent potential application pathways requiring future experimental validation, which will be carried out as part of the Li4Life project^40,41^ (a Horizon Europe research project on sustainable lithium extraction and processing, with UK participation supported by UK Research and Innovation, UKRI).

## Supplementary Material





## Data Availability

The data are available upon request.

## Notes

Raw UV–vis absorbance data for Hammett acidity indicators, pure DESs, and their aqueous solutions (XLSX)
The photo of formulated DES (Figure S1); FTIR spectra for all compounds (Figure S2); Raman spectra for all compounds (Figure S3); ^1^H NMR spectra for all compounds (Figure S4); the optimized secondary structures of DESs at 1:4 and 1:5 ratios (Figure S5); FTIR peak positions for all compounds (Table S1); and Raman peak positions for all compounds (Table S2) (PDF)

## ∥

Department of Metallurgical and Mining Engineering, Universidad Catolica del Norte Facultad de Ingenieria y Ciencias Geologicas, Antofagasta 3580000, Chile
