# Supplementary material for: Thermal Phase Behavior, Structure, Acidity, and Quantum Chemical Insights into Novel Hydroxylamine Hydrochloride (HACl)-Methanesulfonic Acid (MSA) Deep Eutectic Solvents
Source: ACS Omega. 2026 Oct 22;11(43):66871–86. doi: 10.1021/acsomega.6c06790 (PMC13737971; doi:10.1021/acsomega.6c06790)
Supplement: Supplementary file 1 [file ao6c06790_si_002.pdf]

Supplementary Information

Thermal Phase Behaviour, Structure, Acidity, and Quantum Chemical Insights into Novel Hydroxylamine Hydrochloride (HACl)-Methanesulfonic Acid (MSA) Deep Eutectic Solvents

Hosein Ghaedi [a,*], Payam Kalhor [b], Cristian Serrano Araya [a,†], Richard Telford [c], Yousef Ghorbani [a,*]

[a] College of Health and Science, School of Natural Sciences, Department of Chemistry, Joseph Banks Laboratories, University of Lincoln, Green Lane, Lincoln, Lincolnshire LN6 7DL, United Kingdom

[b] School of Chemistry, University of Southampton, Southampton, SO17 1BJ, United Kingdom

[c] School of Chemistry and Biosciences, Faculty of Health and Social Care, University of Bradford, Richmond Road, Bradford, BD7 1DP, United Kingdom

† Present address: Department of Metallurgical and Mining Engineering, Universidad Catolica del Norte Facultad de Ingenieria y Ciencias Geologicas, Antofagasta 3580000, Chile.

*Corresponding authors:

E-mail addresses: hghaedi@lincoln.ac.uk (H.Ghaedi); YGhorbani@lincoln.ac.uk (Y. Ghorbani).

The supplementary information includes the photo of formulated DES (Fig. S1), FTIR spectra for all compounds (Fig. S2), Raman spectra for all compounds (Fig. S3), ^{1}H NMR spectra for all compounds (Fig. S4), the optimised secondary structures of DESs at 1:4 and 1:5 ratios (Fig. S5), FTIR peak positions for all compounds (Table S1), and Raman peak positions for all compounds (Table S2).

List of Figures

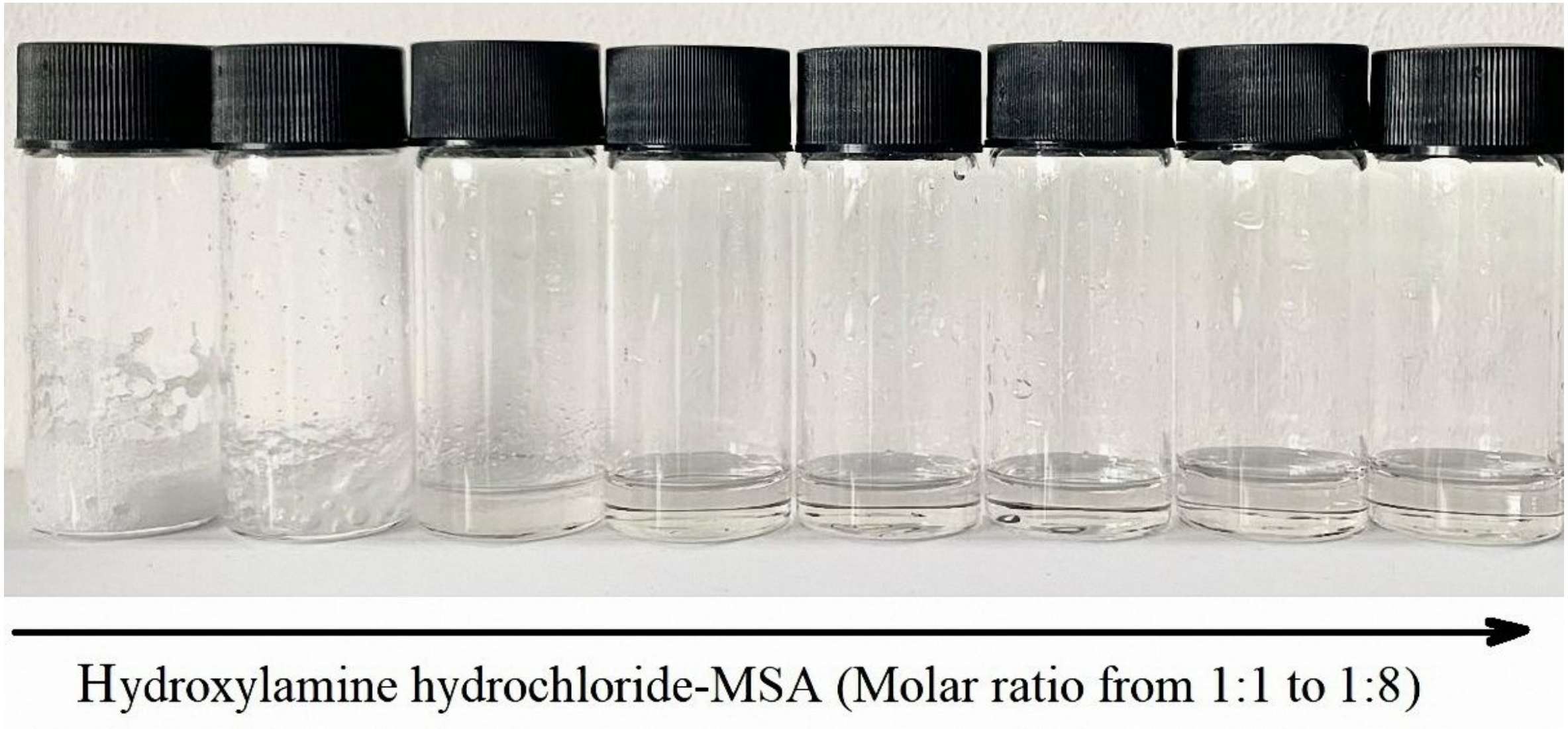

Fig. S1. Photos of formulated HACl-MSA DESs from molar ratios of 1:1 to 1:8.

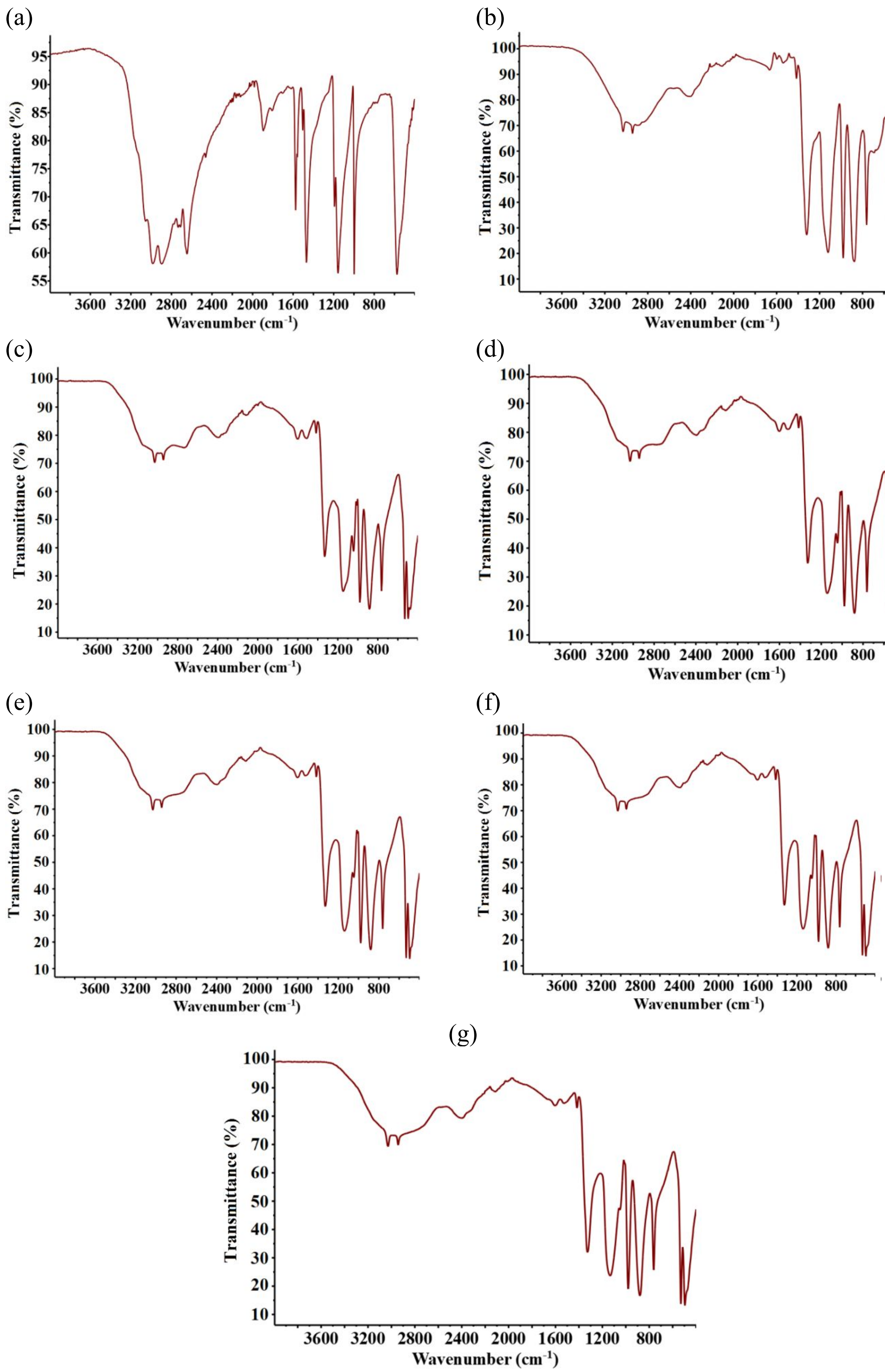

Fig. S2. FTIR spectra of (a) pure HACl, (b) pure MSA, (c) HACl-MSA (1:4), (d) HACl-MSA (1:5), (e) HACl-MSA (1:6), (f) HACl-MSA (1:7), (g) HACl-MSA (1:8).

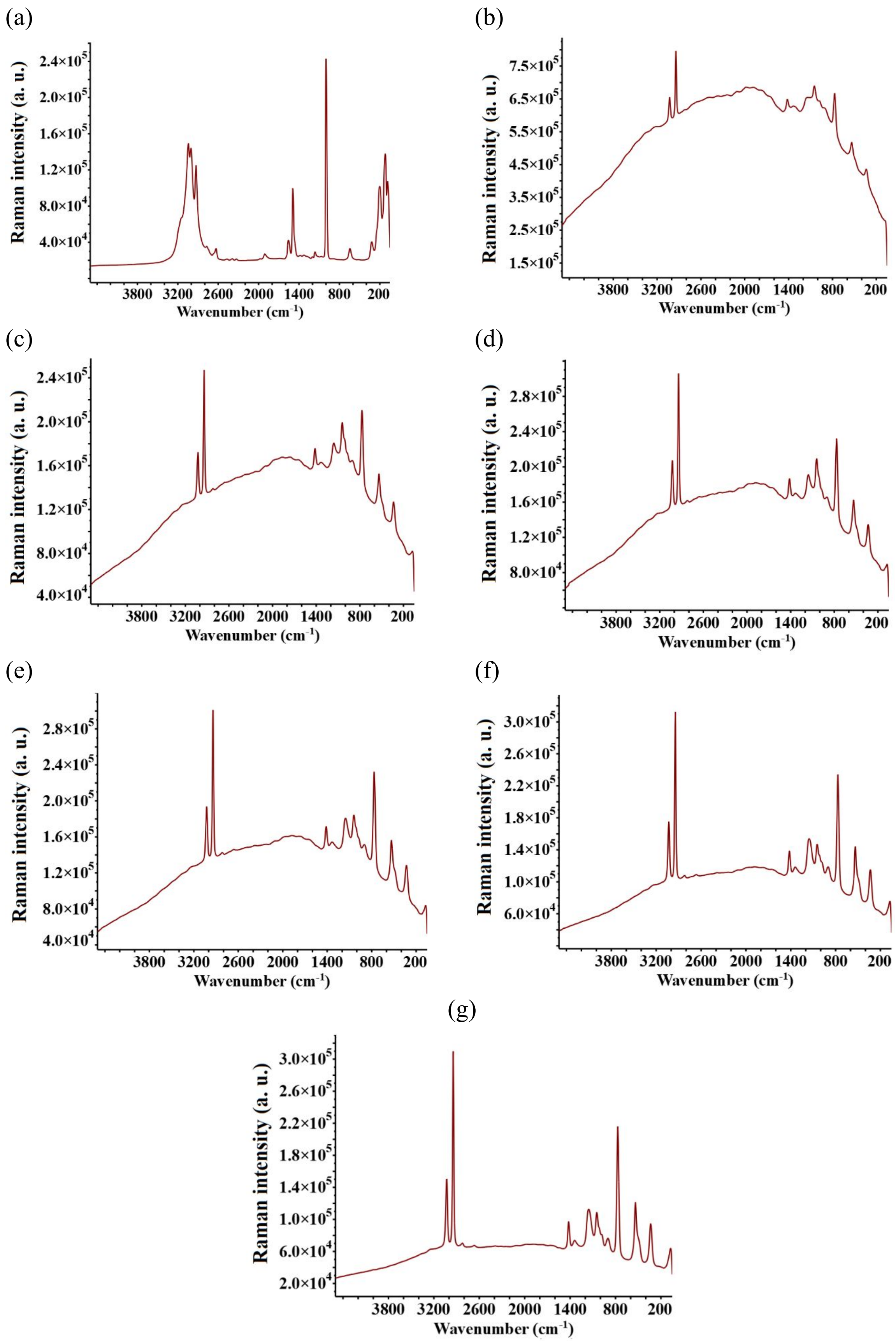

Fig. S3. Raman spectra of (a) pure HACl, (b) pure MSA, (c) HACl-MSA (1:4), (d) HACl-MSA (1:5), (e) HACl-MSA (1:6), (f) HACl-MSA (1:7), (g) HACl-MSA (1:8).

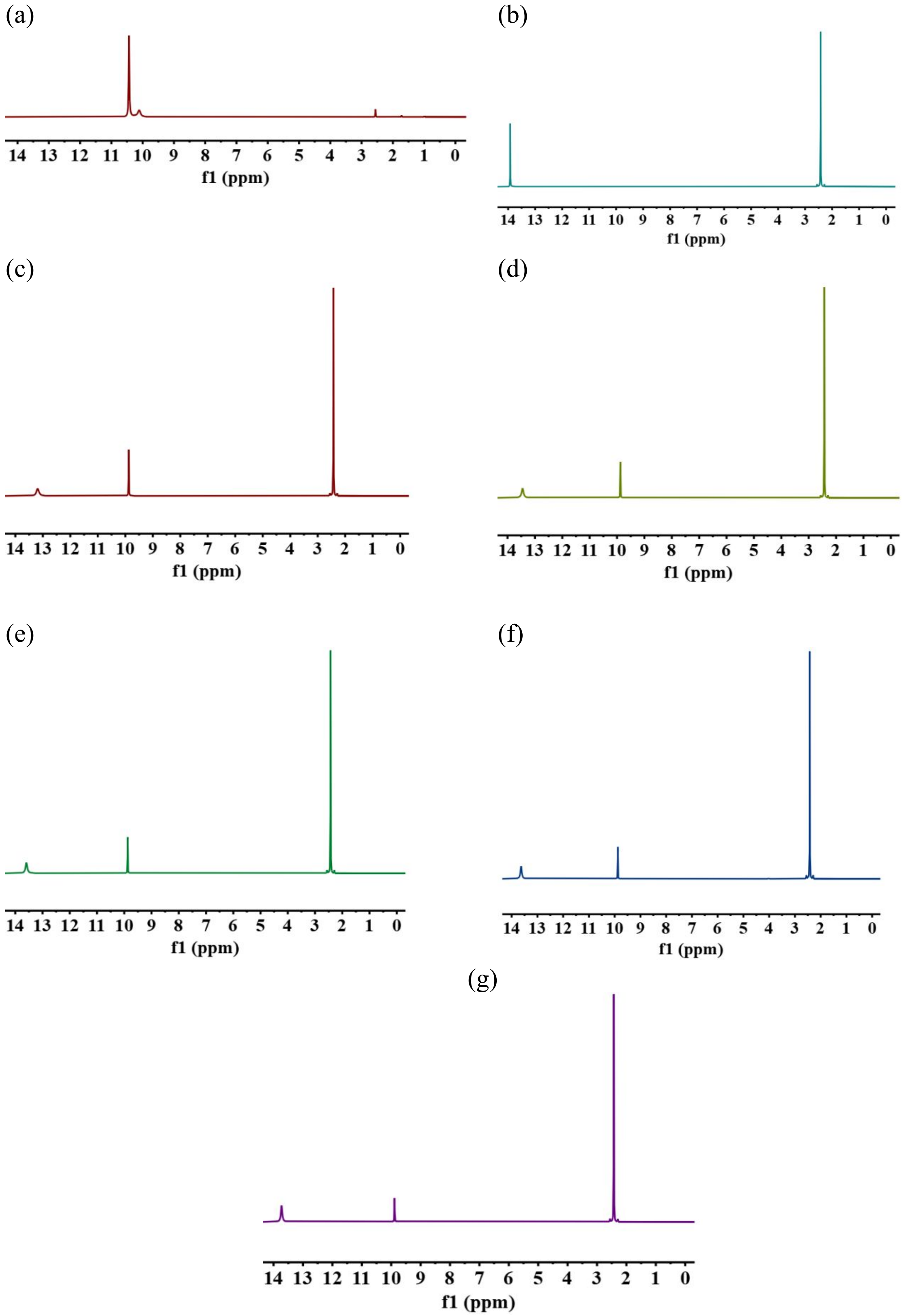

Fig. S4. ^{1}H NMR spectra of (a) pure HACl, (b) pure MSA, (c) HACl-MSA (1:4), (d) HACl-MSA (1:5), (e) HACl-MSA (1:6), (f) HACl-MSA (1:7), (g) HACl-MSA (1:8).

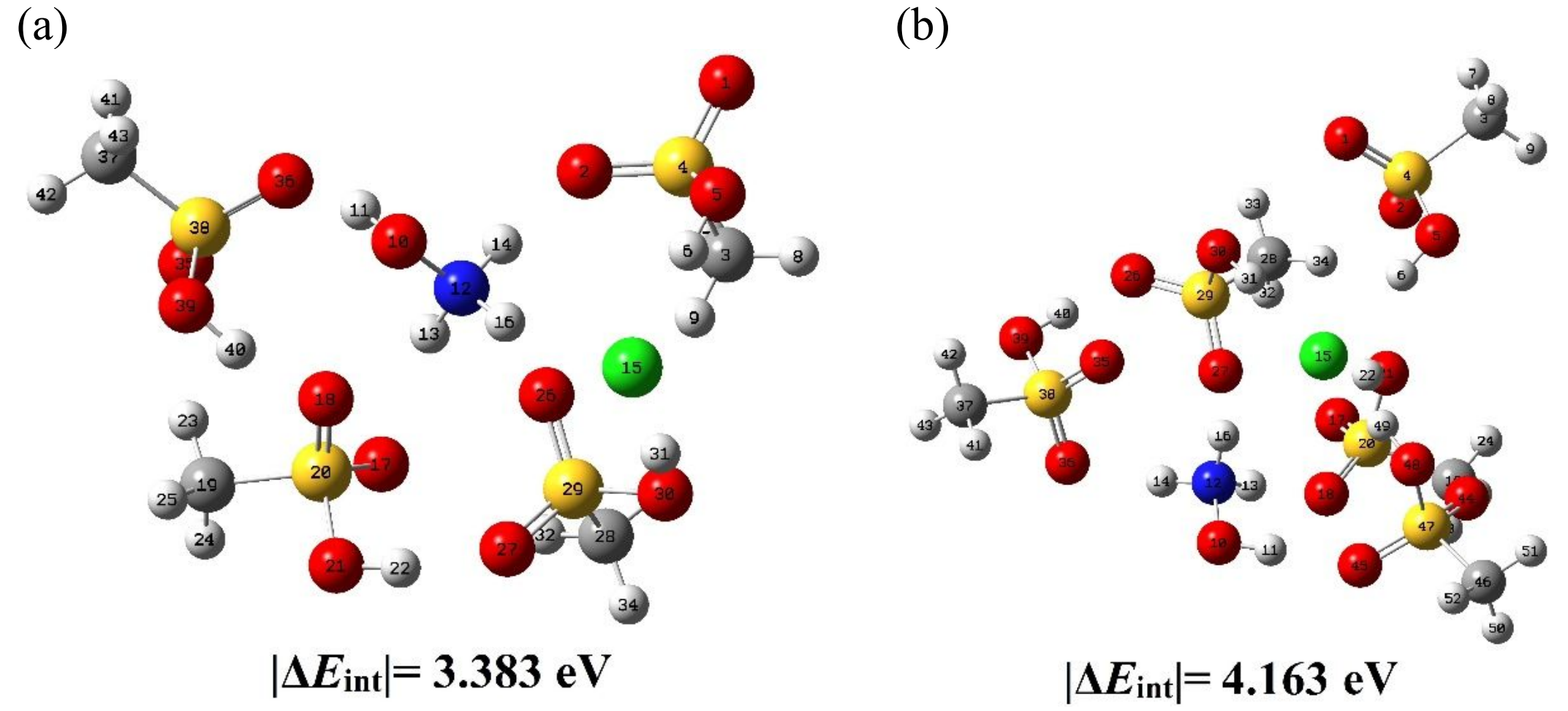

Fig. S5. Optimised geometries of (a) HACl-MSA (1:4) and (b) HACl-MSA (1:5) with their $|\Delta E_{int}|$ values.

List of Tables

Table S1. FTIR peak positions (wavenumber/cm^{-1}) for HACl-MSA DESs and their individual compounds.

HACl-MSA (1:4)	HACl-MSA (1:5)	HACl-MSA (1:6)	HACl-MSA (1:7)	HACl-MSA (1:8)	Pure MSA	Pure HACl
477.14	479.18	495.49	495.49	495.49	493.5	575
497.53	495.49	530.16	530.16	530.16	528	997.1
530.16	530.16	762.61	762.61	762.61	762.6	1156
762.61	762.61	880.88	880.88	878.84	876.8	1190.8
882.91	880.88	978.75	978.75	978.75	980.8	1468
978.75	978.75	1009.34	1009.34	1009.34	1121.5	1504.8
1009.34	1009.34	1046.04	1048.08	1050.12	1319	1576
1041.96	1044.00	1139.84	1137.80	1131.68	1415	1808.7
1145.95	1141.88	1329.47	1327.43	1327.43	1539.5	1894
1331.51	1329.47	1417.15	1417.15	1415.11	1598	1982
1417.15	1417.15	1525.22	1527.26	1527.26	1665.5	2461
1510.95	1515.02	1602.70	1604.74	1604.74	2408	2646.7
1600.66	1600.66	2110.43	2114.51	2116.55	1942	2712
2118.59	2116.55	2395.90	2393.86	2395.90	3030	2734
2393.86	2395.90	2942.37	2942.37	2942.37		2893
2736.42	2748.66	3030.05	3030.05	3030.05		2985
2942.37	2942.37					3054.5
3030.05	3030.05					
3136.08	3138.12					

Table S2. Raman peak positions (wavenumber/cm^{-1}) for HACl-MSA DESs and their individual compounds.

HACl-MSA (1:4)	HACl-MSA (1:5)	HACl-MSA (1:6)	HACl-MSA (1:7)	HACl-MSA (1:8)	Pure HACl	Pure MSA
75.00	72.00	70.50	73.50	72.00	84.00	336
336.00	334.50	331.50	334.50	334.50	118.50	535
537.00	535.50	532.50	537.00	537.00	199.50	769.5
769.50	768.00	766.50	769.50	769.50	321.00	1047
904.50	901.50	900.00	900.00	900.00	643.50	1416
1042.50	1041.00	1041.00	1045.50	1047.00	999.00	2943
1158.00	1156.50	1155.00	1153.50	1155.00	1162.50	3027
1335.00	1336.50	1335.00	1339.50	1339.50	1492.50	3246
1417.50	1416.00	1414.50	1419.00	1419.00	1557.00	3357
2820.00	2821.50	2815.50	2820.00	2821.50	1906.50	
2941.50	2941.50	2941.50	2944.50	2944.50	2632.50	
3027.00	3027.00	3027.00	3030.00	3031.50	2929.50	
3226.50	3226.50	3235.50	3239.50	3244.00	3006.00	
3343.50	3350.00	3352.00	3355.50	3363.00		
